# Supplementary material for: ASPSCR1-TFE3 reprograms transcription by organizing enhancer loops around hexameric VCP/p97
Source: Nat Commun. 2024 Feb 7;15:1165. doi: 10.1038/s41467-024-45280-5 (PMC10850509; doi:10.1038/s41467-024-45280-5)
Supplement: Supplementary file 1 — Supplementary Information [file 41467_2024_45280_MOESM1_ESM.pdf]

ASPSCR1-TFE3 reprograms transcription by organizing enhancer loops around hexameric VCP/p97

Amir Pozner, Li Li, Shiv P. Verma, Shuxin Wang, Jared J. Barrott, Mary L. Nelson, Jamie S. E. Yu, Gian Luca Negri, Shane Colborne, Christopher S. Hughes, Ju-Fen Zhu, Sydney L. Lambert, Lara S. Carroll Kyllie Smith-Fry, Michael G. Stewart, Sarmishta Kannan, Bodrie Jensen, Cini M. John, Saif Sikdar, Hongrui Liu, Ngoc Ha Dang, Jennifer Bourdage, Jinxiu Li, Jeffery M. Vahrenkamp, Katelyn L. Mortenson, John S. Groundland, Rosanna Wustrack, Donna L. Senger, Franz J. Zemp, Douglas J. Mahoney, Jason Gertz, Xiaoyang Zhang, Alexander J. Lazar, Martin Hirst, Gregg B. Morin, Torsten O. Nielsen, Peter S. Shen, Kevin B. Jones

Supplementary Information

	pages
Supplementary Figures with Legends	2 - 15
Supplementary Tables	16 – 29
Supplementary References	29

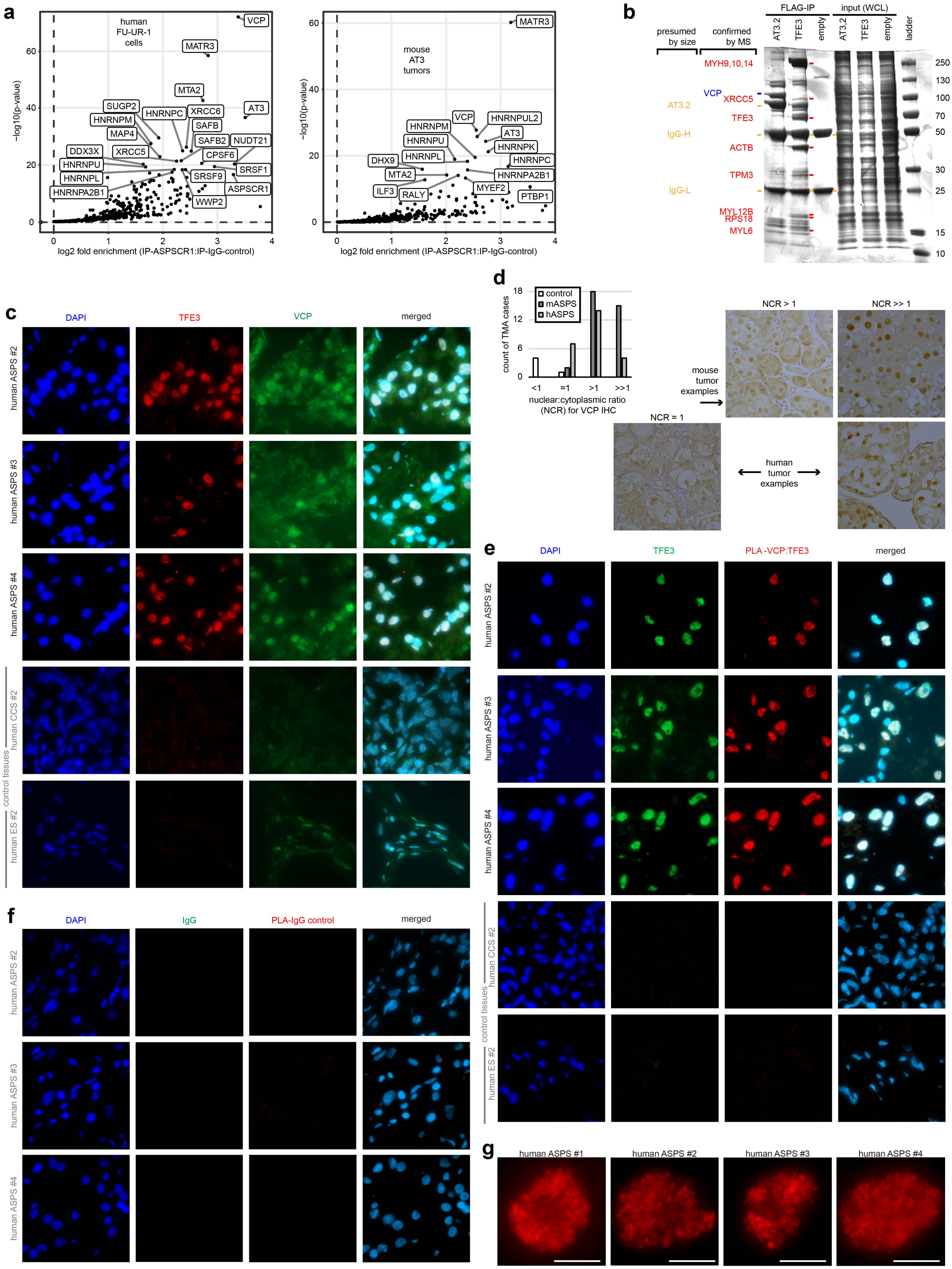
a
human FU-UR-1 cells
mouse AT3 tumors
−log10(p-value)
log2 fold enrichment (IP-ASPSCR1:IP-IgG-control)
VCP
MATR3
MTA2
AT3
XRCC6
SAFB
SAFB2
NUDT21
CPSF6
SRSF1
SRSF9
ASPSCR1
WWP2
SUGP2
HNRNPM
HNRNPC
MAP4
DDX3X
XRCC5
HNRNPU
HNRNPL
HNRNPA2B1
HNRNPUL2
HNRNPK
DHX9
MYEF2
PTBP1
ILF3
RALY
b
presumed by size
confirmed by MS
FLAG-IP
input (WCL)
AT3.2
TFE3
empty
ladder
MYH9,10,14
VCP
XRCC5
AT3.2
TFE3
IgG-H
ACTB
TPM3
IgG-L
MYL12B
RPS18
MYL6
250
130
100
70
50
30
25
15
10
c
DAPI
TFE3
VCP
merged
human ASPS #2
human ASPS #3
human ASPS #4
human CCS #2
human ES #2
control tissues
d
count of TMA cases
control
mASPS
hASPS
nuclear:cytoplasmic ratio (NCR) for VCP IHC
NCR > 1
NCR >> 1
NCR = 1
mouse tumor examples
human tumor examples
e
PLA -VCP:TFE3
f
IgG
PLA-IgG control
g
human ASPS #1
human ASPS #2
human ASPS #3
human ASPS #4

Supplementary Figure 1. ASPSCR1-TFE3 interacts with VCP/p97 in the nucleus of ASPS and RCC tumor cells through its ASPSCR1 portion. (**a**) Identification of the proteins enriched in both human FU-UR-1 cells and mouse AT3-induced tumor tissues by IP-MS of anti-ASPSCR1 IPs from nuclear lysates. (**b**) Coomassie stain of FLAG-IP and whole cell lysate (WCL) input from HEK transfected with TFE3, AT3.2, or empty vector, indicating the MS-identified bands that were visually discrepant between the two IP baits. (**c**) Fluorescence photomicrographs of 5 human tumors, three additional ASPS tumors and two controls with an additional clear cell sarcoma (CCS) and an additional Ewing sarcomas (ES), stained with DAPI, anti-TFE3 (to detect AT3) or anti-VCP immunofluorescent antibodies. (Each image panel represents 100μm square). (**d**) Bar chart of the number of cases staining for variable ratios of nuclear-to-cytoplasmic (NCRs) staining for VCP immunohistochemistry (IHC) on two tissue microarrays (TMAs) for ASPS tumors, one from human tumors and another from mouse tumors. Representative photomicrographs of different ratios of staining from the mouse and human TMAs. (Each image panel represents 100μm square). (**e**) Fluorescence photomicrographs demonstrating the proximity ligation assay (PLA) for the interaction between anti-TFE3 and anti-VCP antibodies in the same three ASPS tumors and two control human tumor tissues as in C. (Each image panel represents 100μm square). (**f**) Fluorescence photomicrographs demonstrating the negative control PLA for the interaction between control antibodies in the three additional human ASPS tumor tissues from **Figure 1**. (Each image panel represents 100μm square). (**g**) Higher magnification photomicrographs of single nuclei from each ASPS tumor, demonstrating the PLA signal character (magnification bars = 5μm).

Supplementary Figure 2

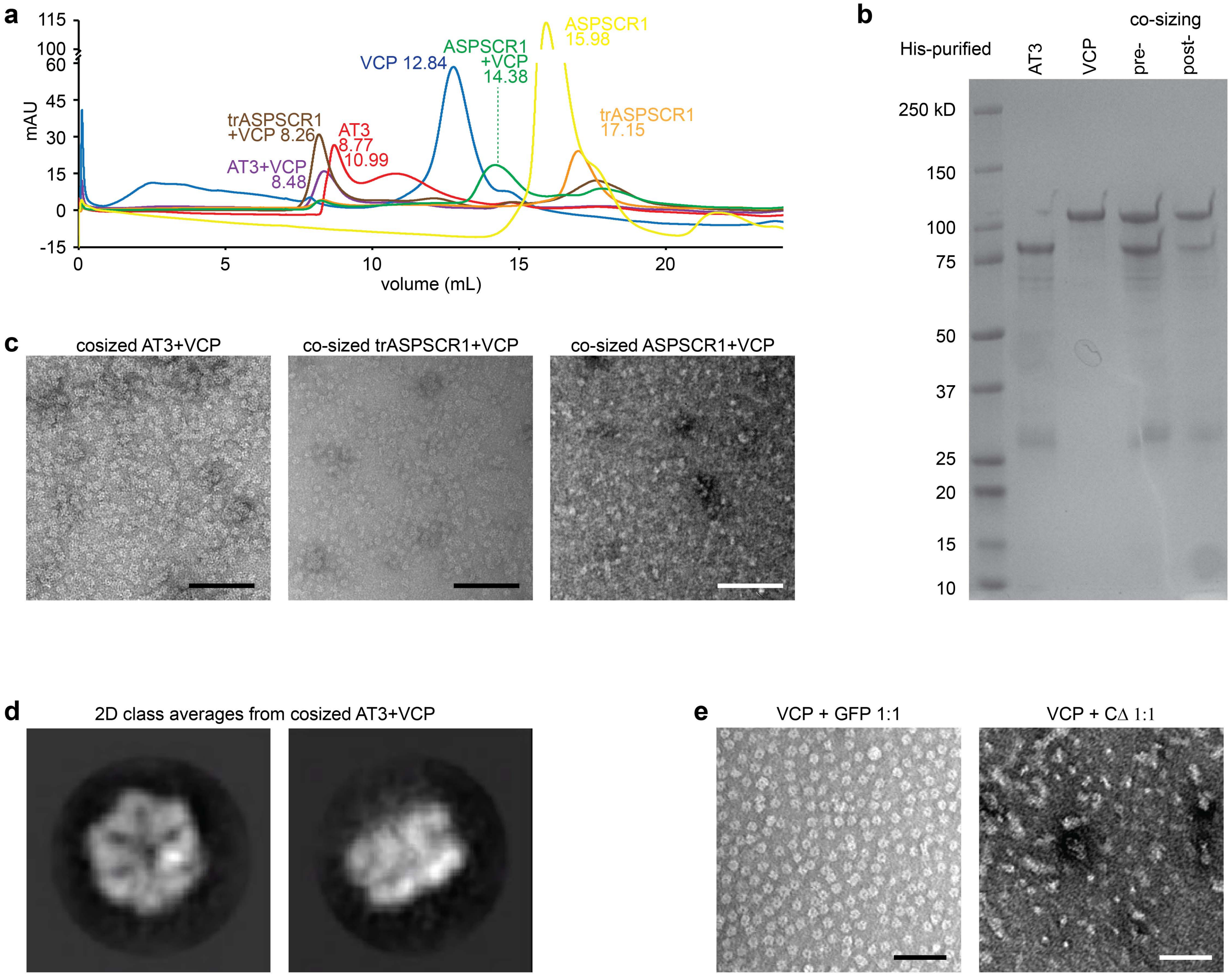

Supplementary Figure 2. ASPSCR1-TFE3 interacts with hexameric assemblies of VCP/p97. (**a**) Co-sizing size exclusion chromatography of each individual purified recombinant protein (ASPSCR1, trASPSCR1, AT3), as well as each after combining with VCP. (**b**) Coomassie stained SDS-PAGE of AT3 and VCP proteins before and after co-sizing and selection of the fraction representing the combination peak (8.49 mL in panel A). (**c**) Negative stain TEM of co-sized fractions of recombinant AT3, trASPSCR1, and ASPSCR1, each with VCP (scale bars = 100nm). (**d**) 2D class averages showing a top or pore view (1,406 particles) and side view (2,301 particles) of VCP hexamers, identified on co-sizing with AT3, but not ASPSCR1 full length (box length and width = 25 nm). (**e**) Negative stain TEM of VCP combined with equimolar GFP control or CΔ (scale bars = 100nm).

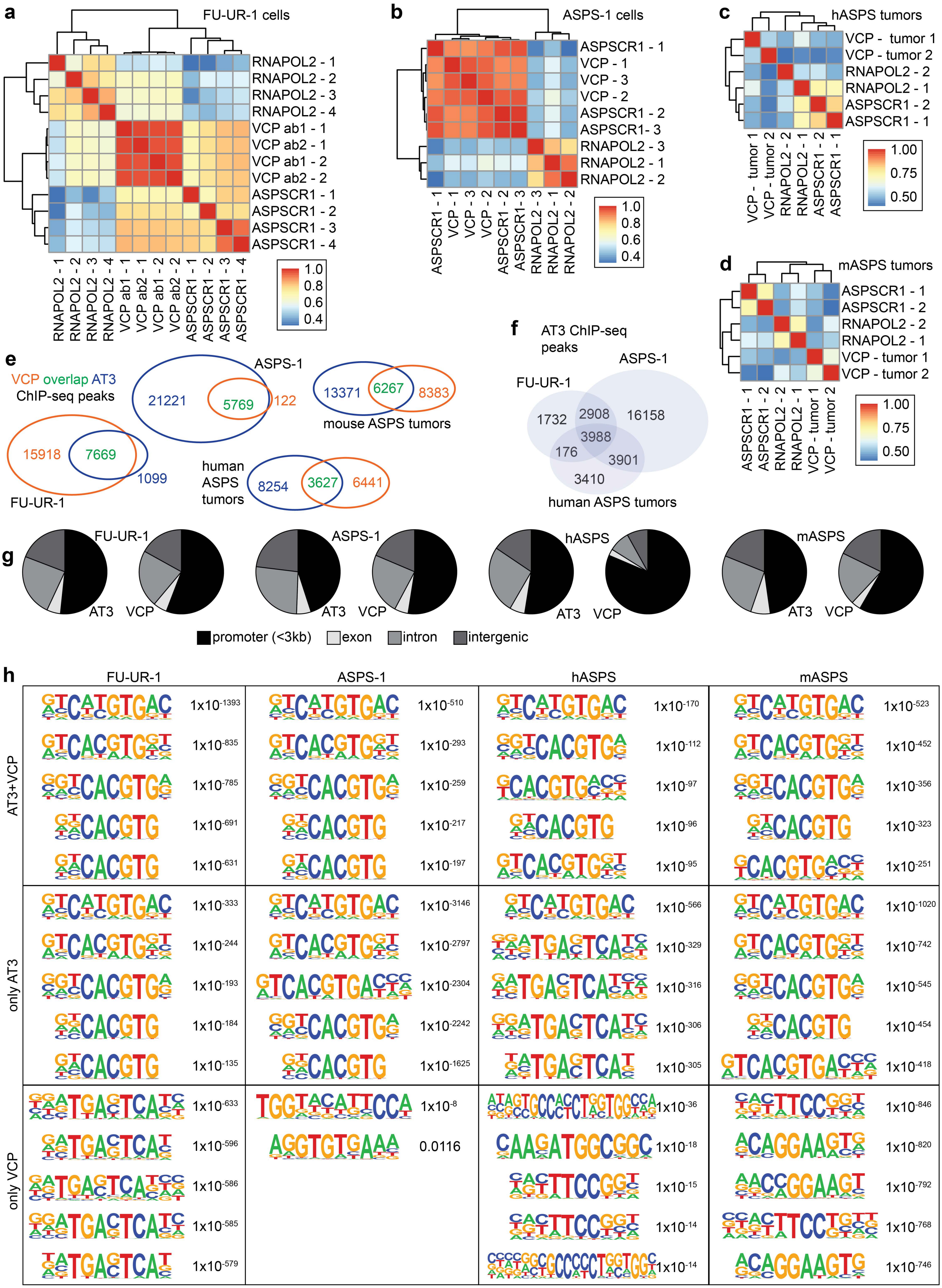
a
FU-UR-1 cells
RNAPOL2 - 1
RNAPOL2 - 2
RNAPOL2 - 3
RNAPOL2 - 4
VCP ab1 - 1
VCP ab2 - 1
VCP ab1 - 2
VCP ab2 - 2
ASPSCR1 - 1
ASPSCR1 - 2
ASPSCR1 - 3
ASPSCR1 - 4
b
ASPS-1 cells
ASPSCR1 - 1
VCP - 1
VCP - 3
VCP - 2
ASPSCR1 - 2
ASPSCR1- 3
RNAPOL2 - 3
RNAPOL2 - 1
RNAPOL2 - 2
c
hASPS tumors
VCP - tumor 1
VCP - tumor 2
RNAPOL2 - 2
RNAPOL2 - 1
ASPSCR1 - 2
ASPSCR1 - 1
d
mASPS tumors
ASPSCR1 - 1
ASPSCR1 - 2
RNAPOL2 - 2
RNAPOL2 - 1
VCP - tumor 1
VCP - tumor 2
e
VCP overlap AT3
ChIP-seq peaks
ASPS-1
21221
5769
122
13371
6267
8383
mouse ASPS tumors
15918
7669
1099
FU-UR-1
human ASPS tumors
8254
3627
6441
f
AT3 ChIP-seq peaks
ASPS-1
FU-UR-1
1732
2908
16158
3988
176
3901
3410
human ASPS tumors
g
FU-UR-1
ASPS-1
hASPS
mASPS
AT3
VCP
promoter (<3kb)
exon
intron
intergenic
h
FU-UR-1
ASPS-1
hASPS
mASPS
AT3+VCP
only AT3
only VCP
1x10-1393
1x10-835
1x10-785
1x10-691
1x10-631
1x10-510
1x10-293
1x10-259
1x10-217
1x10-197
1x10-170
1x10-112
1x10-97
1x10-96
1x10-95
1x10-523
1x10-452
1x10-356
1x10-323
1x10-251
1x10-333
1x10-244
1x10-193
1x10-184
1x10-135
1x10-3146
1x10-2797
1x10-2304
1x10-2242
1x10-1625
1x10-566
1x10-329
1x10-316
1x10-306
1x10-305
1x10-1020
1x10-742
1x10-545
1x10-454
1x10-418
1x10-633
1x10-596
1x10-586
1x10-585
1x10-579
1x10-8
0.0116
1x10-36
1x10-18
1x10-15
1x10-14
1x10-14
1x10-846
1x10-820
1x10-792
1x10-768
1x10-746

Supplementary Figure 3. ASPSCR1-TFE3 and VCP interact on chromatin by ChIP-seq. (**a**) Pearson correlation heatmap of biological replicates of ChIP-seq using antibodies against RNAPOL2, VCP, and ASPSCR1 amino terminus for AT3 in FU-UR-1 cells, (**b**) ASPS-1 cells, (**c**) 2 human ASPS tumors, and (**d**) two mouse AT3-initiated ASPS tumors. (**e**) Venn diagrams indicating the overlap of called peaks for AT3 with VCP in different models. (**f**) Venn diagrams indicating the overlap of called peaks for AT3 between the two human cell lines and human ASPS tumors. (**g**) Annotation pie charts for AT3 and VCP called peaks genome-wide with respect to genes between the 4 different systems. (**h**) Representation of the 5 most common known motifs in each subset of peaks from each group in Fig. 3a, peaks called for both AT3 and VCP, those called only for AT3, and those called only for VCP, with listed p-values.

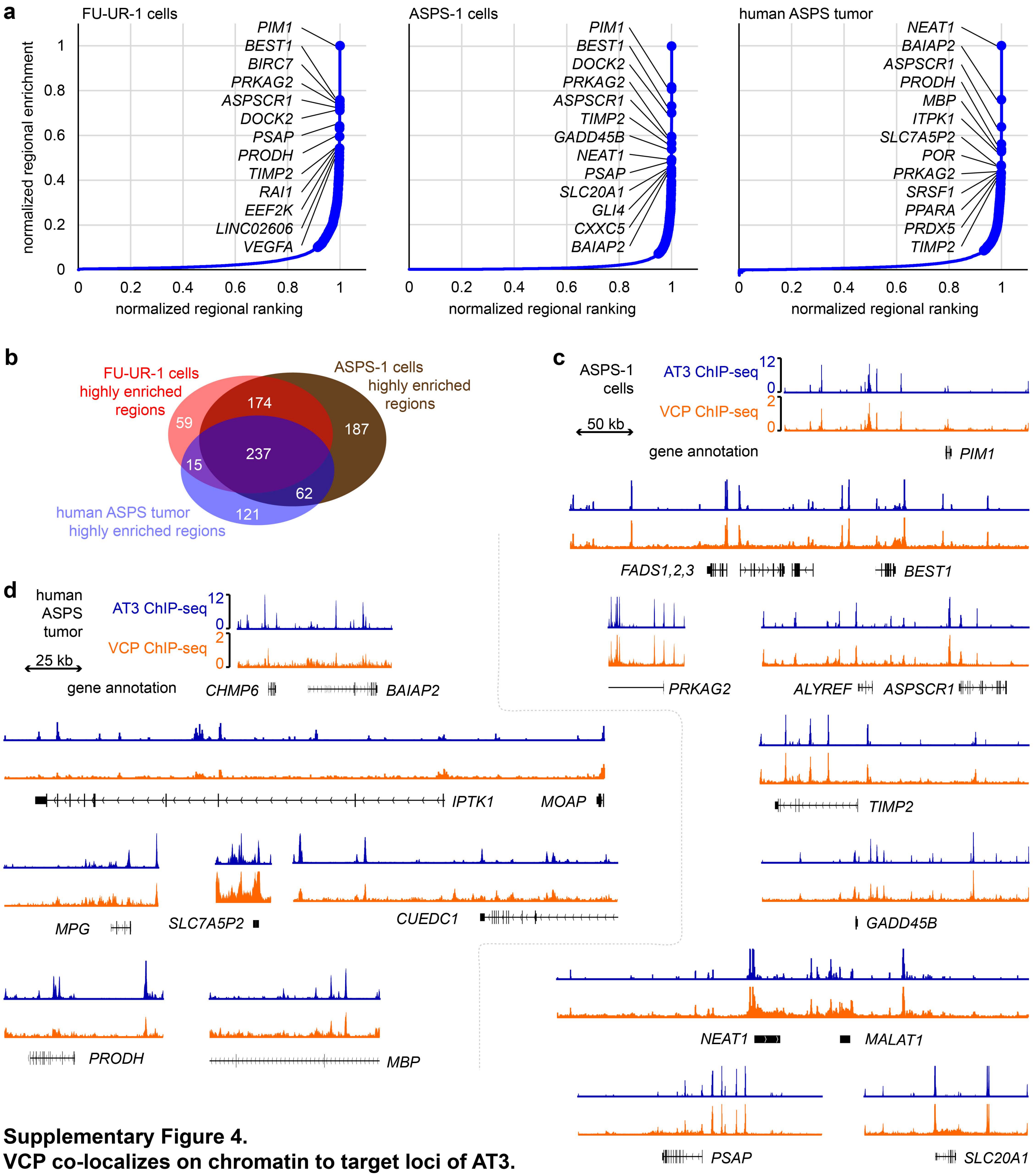

Supplementary Figure 4.
VCP co-localizes on chromatin to target loci of AT3.
(**a**) Plots of the normalized enrichment for AT3 ChIP-seq over the normalized ranking of enrichment for AT3 ChIP-seq in regions stitched together by stringently called peaks within a 40kb gap of each other for FU-UR-1 cells, ASPS-1 cells, and human ASPS tumor samples. All regions above the slope inflection point of 1 were designated highly enriched regions (and represented approximately the top 5 to 6% of regions). (**b**) Venn diagram of the location-overlapping highly enriched AT3 regions in human FU-UR-1 cells, ASPS-1 cells, and human ASPS tumors. (**c**) Representative tracks from highly enriched stitched regions, showing detailed overlap of AT3 and VCP ChIP-seq enrichment for the ASPS-1 cell line. (**d**) Representative tracks from highly enriched stitched regions in human ASPS tumors.

Supplementary Figure 5

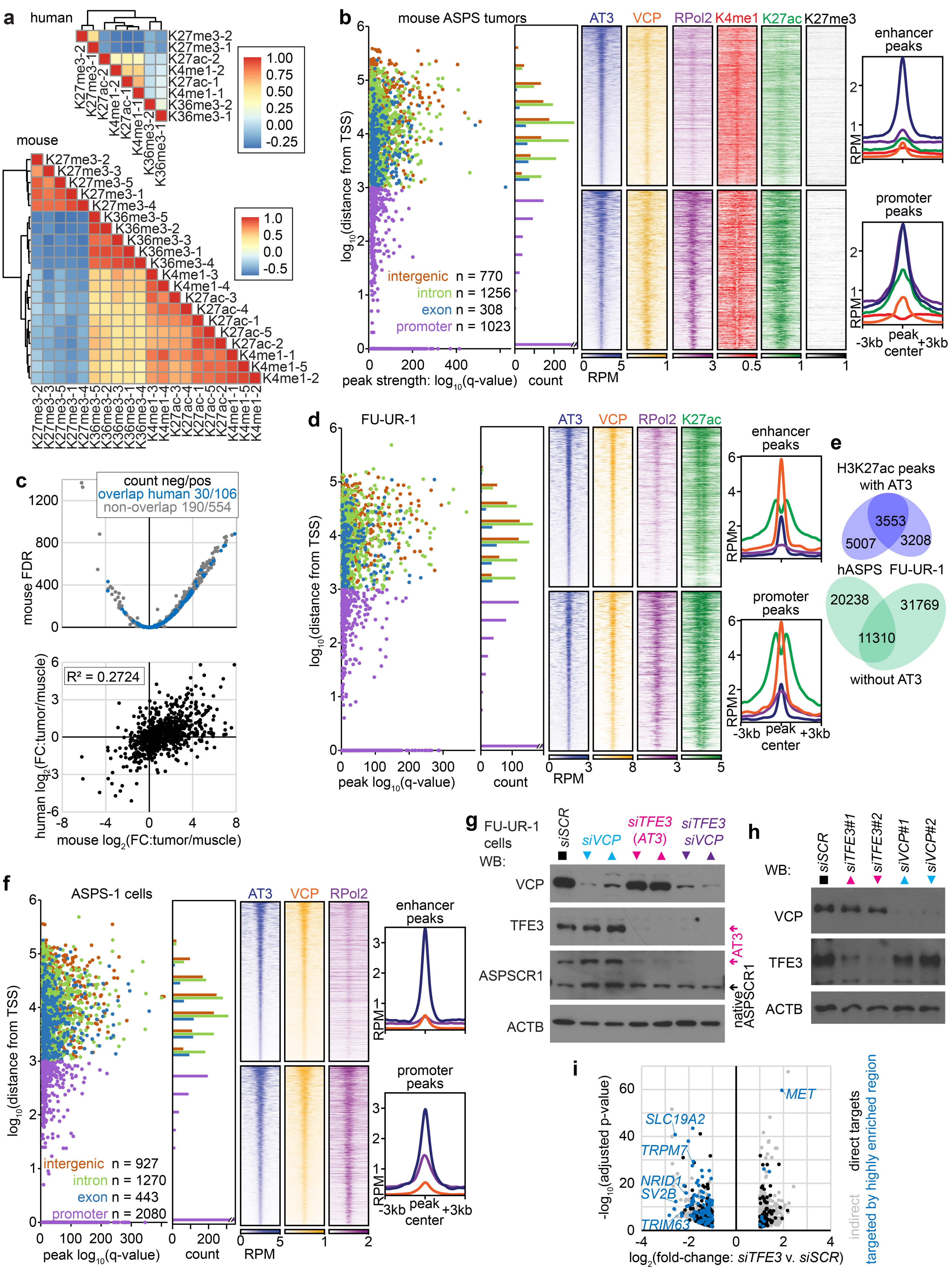

Supplementary Figure 5. AT3:VCP interaction localizes to the promoters and enhancers of target genes. (**a**) Pearson correlation heatmaps for the varied histone marks native ChIP-seq experiments performed on two human ASPS tumors and 5 mouse AT3-initiated ASPS tumors. (**b**) Graph and histogram of the annotations for all AT3 ChIP-seq peaks called in mouse ASPS tumors within the highly enriched regions (defined in an algorithm similar to that for human tumors in Supplemental Figure 5A). Heatmaps of reads per million (RPM) for distal (putative enhancer) and proximal promoter peaks, as well as enrichment plots for the same. (**c**) The mean differential expression of the nearest gene targets of highly enriched AT3 regions in mouse tumors compared to mouse muscle samples by RNAseq (GSE54729, FDR = false discovery rate q-value), noting the genes also annotated in human highly enriched AT3 regions and showing correlation between differential expression in human compared to mouse tumors over muscle. (**d**) Similar data for the FU-UR-1 cell line. (**e**) Venn diagrams showing overlap between human ASPS tumors and FU-UR-1 cells for H3K27ac ChIP-seq peaks genome wide that intersect with AT3 or do not. (**f**) Similar data for the ASPS-1 cell line. (**g**) Western blots (WBs) demonstrating effectiveness of knock-down by 2 different siRNAs against each of VCP, TFE3 (for AT3, as there is no native TFE3 in this male cell line), or both in human FU-UR-1 cells. (SCR = scrambled control. Triangles pointing up or down represent the siRNA#1 and siRNA#2 for each target transcript.) (**h**) WBs demonstrating modest knock-down of AT3 and VCP in ASPS-1 cells with the same siRNAs. (**i**) Differential gene expression following 48-hour siRNA depletion of AT3 in FU-UR-1 cells. Blue dots represent genes annotated by highly enriched regions (from **Supplementary Figure 4a**). Genes noted in black are those annotated by AT3 peaks generally. Gray dots denote indirect target genes, without associated AT3 ChIP-seq peaks.

Supplementary Figure 6

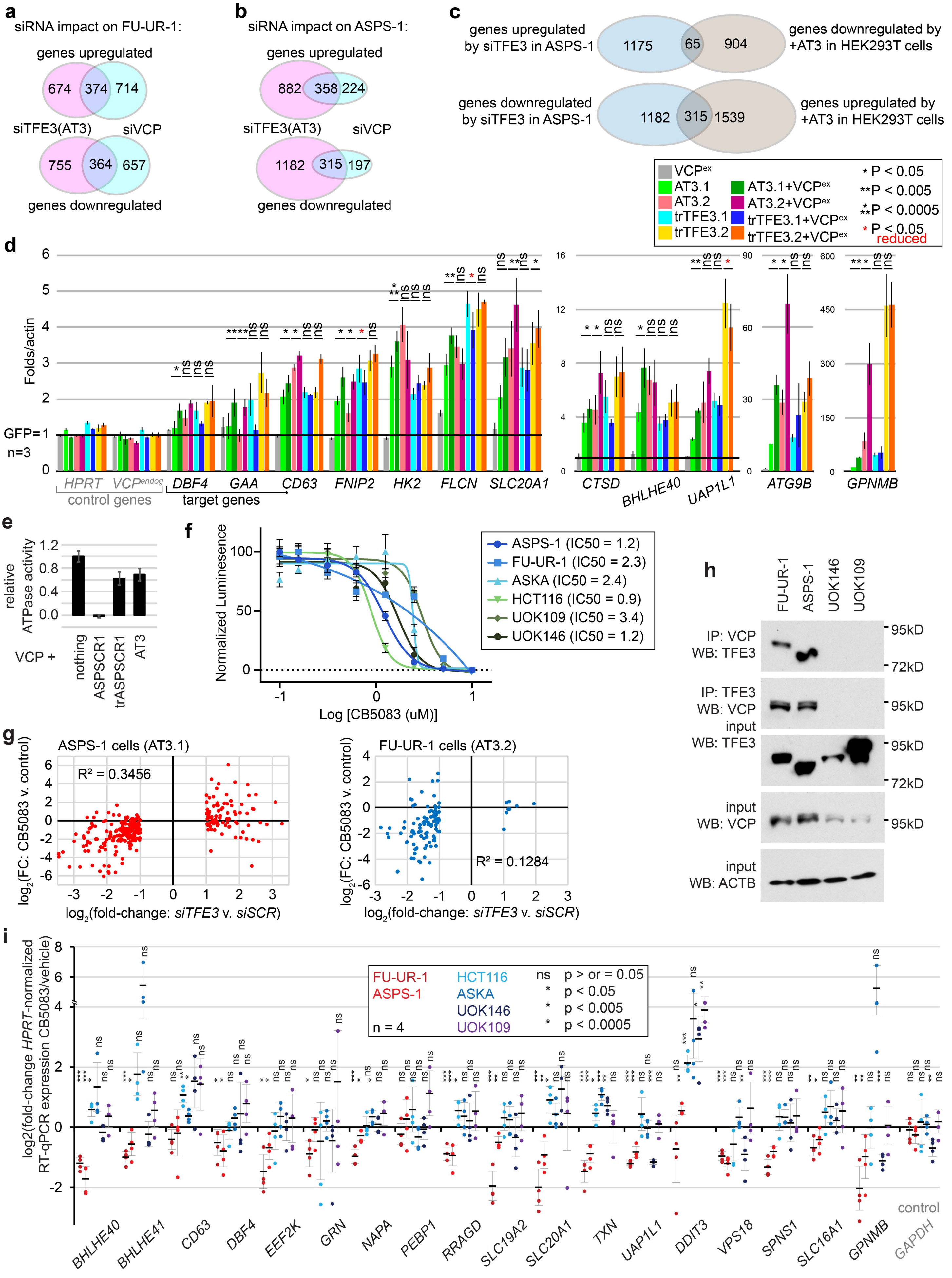

Supplementary Figure 6. VCP presence, hexamerization, and ATPase activity impact AT3-related transcription. (**a**) Venn diagram of overlap of whole transcriptome shifts between FU-UR-1 cells exposed for to *siTFE3* or *siVCP* (pooled from two siRNAs each and n = 3 biological replicates for each siRNA, p-adjusted < 0.05). (**b**) The same Venn diagram for ASPS-1 cells. (**c**) Overlap of significant genes changed in RNA-seq in ASPS-1 cells or HEK293T cellswith or without AT3 present. (**d**) Relative expression by RTqPCR of target genes in HEK293T cells transfected with exogenous VCP overexpression (VCPex) added to AT3.1, AT3.2, or truncated variants of TFE3 corresponding to the portions included in AT3.1 and AT3.2. Each of the statistical notations compares the addition of overexpression of VCP with the transcription factor to the transcription factor alone. (**e**) Relative ATPase activity of recombinant VCP with added recombinant ASPSCR1 (which disassembles hexamers), truncated ASPSCR1 (the portion from AT3, which does not disassemble hexamers), or AT3. (**f**) Proliferation/viability assay with Cell Titer Glo for ASPS-1, FU-UR-1, ASKA (synovial sarcoma), HCT116 (colorectal carcinoma), UOK109 (RCC with *NONO-TFE3* fusion), or UOK146 (RCC with *PRCC-TF*E3 fusion) cells showing decreasing viable cell mass after 48 hours of increasing concentrations of CB-5083, used to determine an appropriate concentration for later expression related assays, at approximately the 50 percent inhibitory concentration (n = 4). (**g**) Correlation of log-transformed fold-changes in gene expression determined by RNA-seq after CB-5083 or vehicle or pooled results of siRNA to deplete AT3 or control. These are shown for genes defined as direct targets of highly enriched regions of AT3:VCP ChIP-seq peaks and as differentially expressed at least 2-fold and significantly by depletion of AT3 over control. Each was applied for 48 hours. CB-5083 experiments had n = 2 sample size for FU-UR-1 and n = 3 for each group for ASPS-1. The siRNA samples had n = 3 for each group, but pooled results from two different siRNAs that led to depletion of AT3. (**h**) Western blots after immunoprecipitation or 10% of input for FU-UR-1 and ASPS-1 cells, as well as two RCC cell lines that express other TFE3 fusions, but do not interact with VCP. (**i**) Expression determined by RT-qPCR after exposing 6 cell lines to CB5083 for 48 hours, normalized each against HPRT expression, demonstrating the decreased expression of most AT3:VCP targets in ASPS-1 and FU-UR-1, but the opposite effect in most other cell lines, even those expressing other TFE3 fusions.

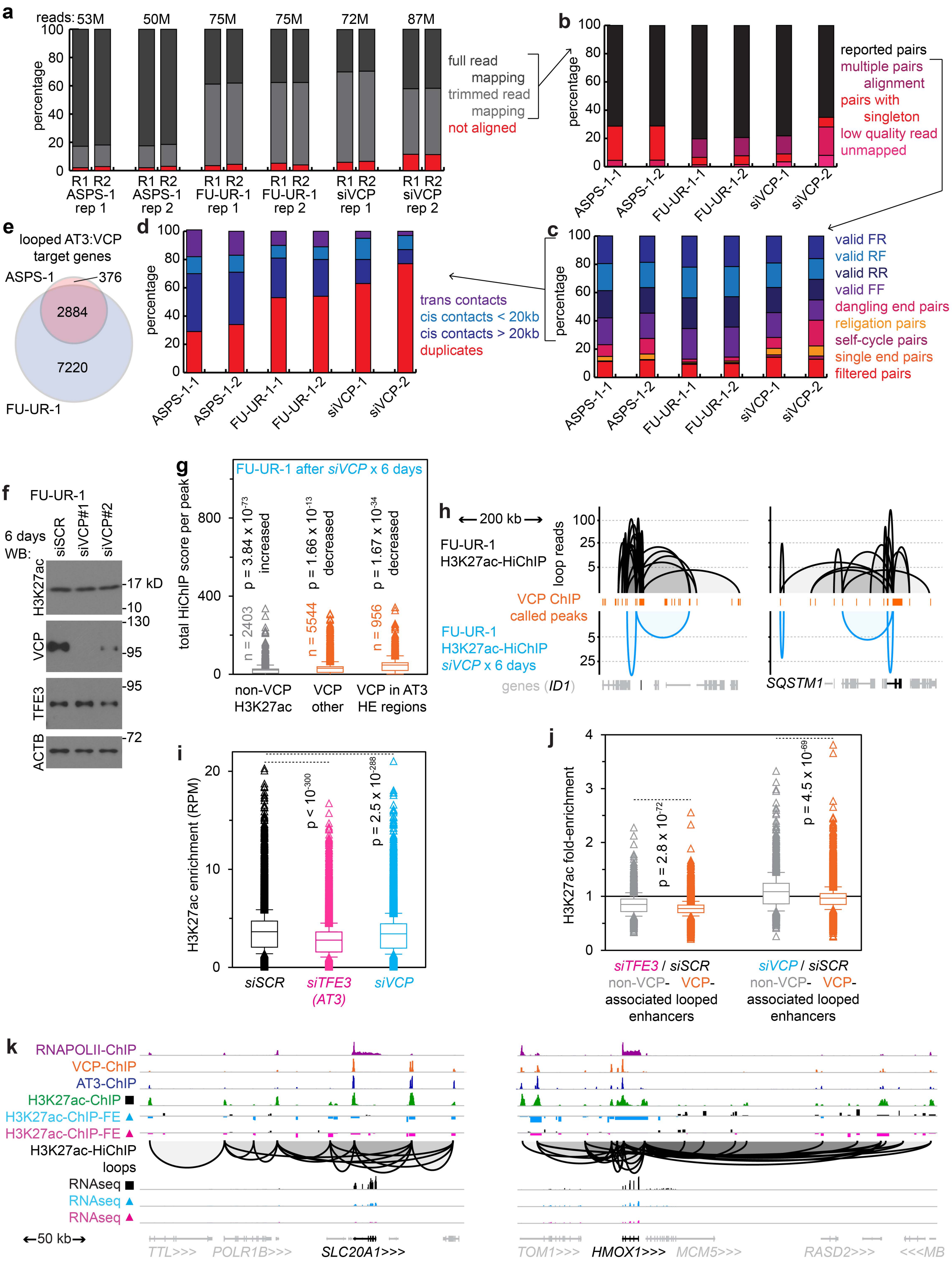
a
reads: 53M 50M 75M 75M 72M 87M
percentage
R1 R2 ASPS-1 rep 1
R1 R2 ASPS-1 rep 2
R1 R2 FU-UR-1 rep 1
R1 R2 FU-UR-1 rep 2
R1 R2 siVCP rep 1
R1 R2 siVCP rep 2
full read mapping
trimmed read mapping
not aligned
b
percentage
ASPS-1-1 ASPS-1-2 FU-UR-1-1 FU-UR-1-2 siVCP-1 siVCP-2
reported pairs
multiple pairs alignment
pairs with singleton
low quality read
unmapped
c
percentage
valid FR
valid RF
valid RR
valid FF
dangling end pairs
religation pairs
self-cycle pairs
single end pairs
filtered pairs
d
percentage
trans contacts
cis contacts < 20kb
cis contacts > 20kb
duplicates
e
looped AT3:VCP target genes
ASPS-1
376
2884
7220
FU-UR-1
f
FU-UR-1
6 days WB:
siSCR siVCP#1 siVCP#2
H3K27ac -17 kD -10
VCP -130 -95
TFE3 -95
ACTB -72
g
FU-UR-1 after siVCP x 6 days
total HiChIP score per peak
p = 3.84 x 10^-73 increased
p = 1.66 x 10^-13 decreased
p = 1.67 x 10^-34 decreased
n = 2403
n = 5544
n = 956
non-VCP H3K27ac
VCP other
VCP in AT3 HE regions
h
200 kb
FU-UR-1 H3K27ac-HiChIP
loop reads
VCP ChIP called peaks
FU-UR-1 H3K27ac-HiChIP siVCP x 6 days
genes (ID1)
SQSTM1
i
H3K27ac enrichment (RPM)
p < 10^-300
p = 2.5 x 10^-288
siSCR
siTFE3 (AT3)
siVCP
j
H3K27ac fold-enrichment
p = 2.8 x 10^-72
p = 4.5 x 10^-69
siTFE3 / siSCR non-VCP- VCP- associated looped enhancers
siVCP / siSCR non-VCP- VCP- associated looped enhancers
k
RNAPOLII-ChIP
VCP-ChIP
AT3-ChIP
H3K27ac-ChIP ■
H3K27ac-ChIP-FE ▲
H3K27ac-ChIP-FE ▲
H3K27ac-HiChIP loops
RNAseq ■
RNAseq ▲
RNAseq ▲
←50 kb→
TTL>>> POLR1B>>> SLC20A1>>>
TOM1>>> HMOX1>>> MCM5>>> RASD2>>> <<<MB

Supplementary Figure 7. Chromatin conformation in the form of enhancer loops depend on the AT3:VCP interaction. (**a**) Quality reports for six H3K27ac-HiChIP experiments, demonstrating the percentage and number of reads mapped per R1 and R2 tag as full reads or trimmed reads, as well as (**b**) the percentage of those read pairs that were reported or failed for the listed reasons. (**c**) The percentage of reported reads that oriented forward (F) or reverse (R), among valid pairs versus failed pairing reasons. (**d**) The distribution of trans, short or long cis, and duplicate (filtered out) contacts from the valid pairs of reported reads, per HiChIP. (**e**) Venn diagram depicting the overlap between looped target genes from **Fig. 6a-b** in the two models. (**f**) Western blots (WB) of H3K27ac and VCP, as well as controls of AT3 (TFE3) and ACTB, after prolonged exposure of FU-UR-1 cells to siSCR control or either of two siVCPs. (**g**) Box plots of the number of HiChIP loop reads per peak in FU-UR-1 cells exposed to siVCP for 6 days, by categories defined in and compared to a (2-tailed heteroschedastic t-test p-values). (**h**) H3K27ac-HiChIP loops in baseline FU-UR-1 cells or after 6 days of siVCP, with reference called peak positions for VCP ChIP. (**i**) Box plots (mean, 25th to 75th percentile, +/- standard deviation, and individual outliers, 2-tailed heteroschedastic t-test p-values) comparing H3K27ac ChIP-seq enrichment (reads per million, RPM) after 48 hours of exposure to siRNAs targeting scrambled control, TFE3 (AT3), or VCP, in FU-UR-1 cells (n = 3 for each of 2 siRNAs for each target, p-value represents a 2-tailed paired t-test showing reduced enrichment after siTFE3 or siVCP). (**j**) Box plots comparing all non-VCP-associated H3K27ac HiChIP loops to all VCP-peak-associated H3K27ac HiChIP loops for fold-enrichment for H3K27ac ChIP-seq after knock-down of AT3 or VCP, each compared to scrambled control. (**k**) Example tracks of H3K27ac ChIP-seq after knock-down of VCP or AT3 by siRNA or scrambled control. Track scales are matched between all the RNA-seq and H3K27ac-fold enrichments (FE). Autoscaling rendered ranges of RNAPOL2: 0 to 9, VCP: 0 to 15, AT3: 0 to 9, H3K27ac: 0 to 11, H3K27ac-FE: -1 to +1. HiChIP and RNA-seq read depths (both graphed on logarithmic scale) range up to 39 and 17 for HMOX1, and 32 and 16 for SLC20A1, each respectively.

Supplementary Figure 8

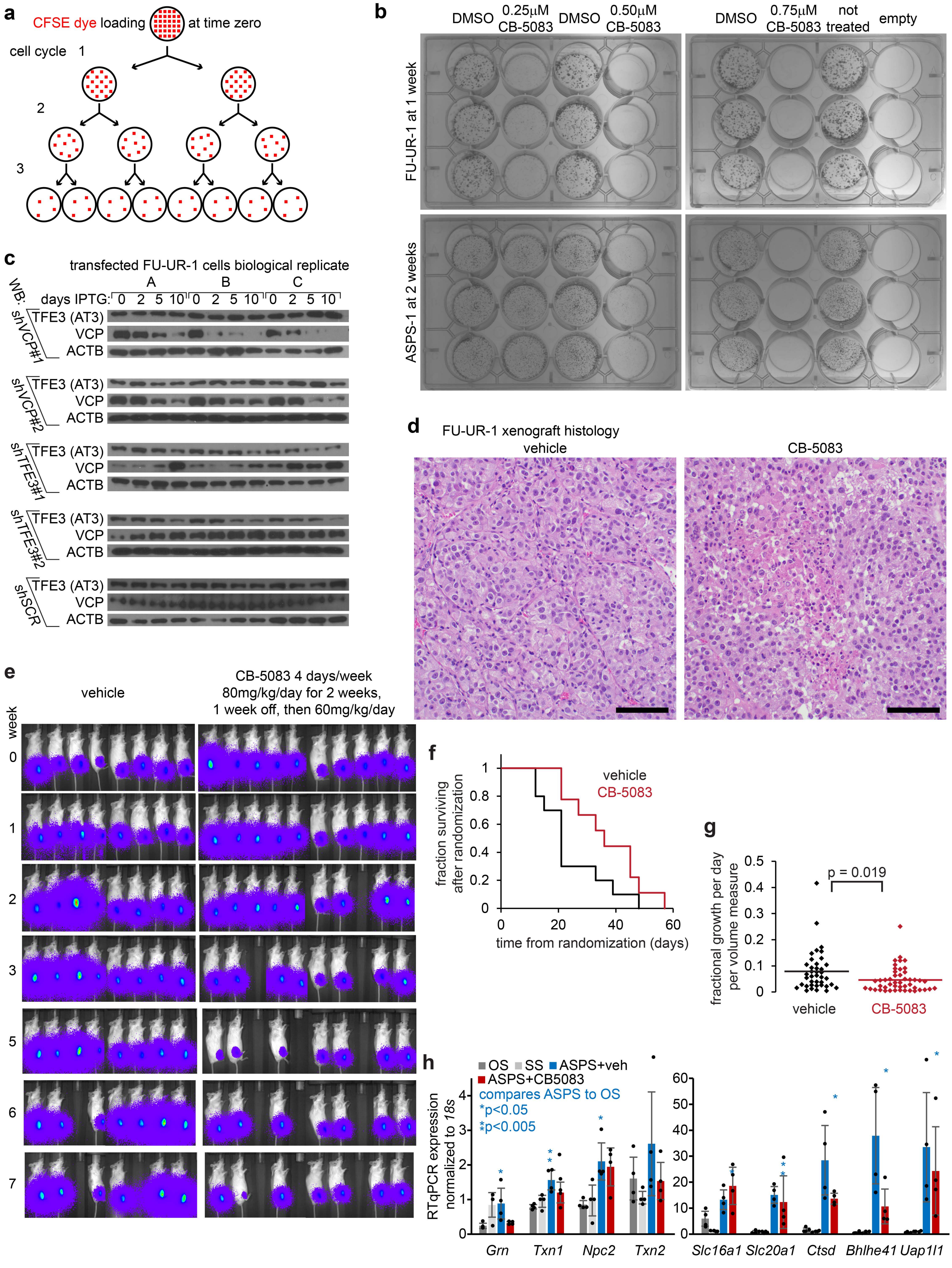

Supplementary Figure 8. AT3:VCP is a targetable functional dependency in cancer cells. (**a**) Schematic of CSFE dye depletion as a measure of proliferation. (**b**) Triplicate colony formation assays in FU-UR-1 and ASPS-1 cells in increasing concentrations of CB-5083 versus control DMSO to the left of each. (**c**) WBs demonstrating the modest depletion of AT3 or VCP by application of IPTG-inducible lentiviral vectors for shRNAs for the same sequences as siRNAs #1 and #2 for each target. These WBs represent whole cell lysates of FU-UR-1 cells after infection, selection, and development of 3 biological replicates for each shRNA. Each population of cells presented is after no IPTG or 2, 5, or 10 days of IPTG in culture. (**d**) Photomicrograph of hematoxylin and eosin stained sections of tumors that developed from FU-UR-1 xenografted cells into NRG mice, subsequently treated with CB-5083 or control vehicle (bar = 100mm.) (**e**) Raw luciferase FLUX data from mice implanted with an ASPS patient-derived xenograft, treated with CB-5083 or vehicle control. (**f**) Kaplan-Meier survival plots of mice randomized to treatment with vehicle (n = 10) or 50mg/kg daily 4 days per week CB-5083 by oral gavage (n = 9). (Mean time to morbidity and standard deviation 24.6 ± 11.9 days compared to 36.4 ± 12.4, respectively; two-tailed homoschedastic t-test, p = 0.034) (**g**) Fractional growth rate per day calculated from measurements at each weekly imaging for each tumor (n = 47, CB-5083; n = 39, vehicle; 2-tailed t-test p-value noted). (**h**) RT-qPCR for target genes (defined as being mouse AT3-ChIP-seq targeted and having reduced expression of homologues in both FU-UR-1 and ASPS-1 human cells upon siRNA depletion of AT3) in control tumors (OS = osteosarcoma, SS = synovial sarcoma) or genetically induced mouse ASPS tumors treated with vehicle or CB-5083 for 50mg/kg for 4 days prior to harvest. (n = 4 tumors per group per gene; homoschedastic 2-tailed t-test p-values noted, comparing vehicle-treated ASPS tumors to OS.)

Supplementary Table 1. Enrichr Reactome pathway analysis for Fig. 4h.

Reactome	Overlap	p-value	Odds Ratio	Combined Score
ASPS-1 AT3 direct target genes				
Activation Of Pre-Replicative Complex R-HSA-68962	6 out of 32	5.84E-07	24.15	346.65
Cell Cycle, Mitotic R-HSA-69278	19 out of 523	9.42E-07	4.13	57.36
Cell Cycle R-HSA-1640170	20 out of 654	6.60E-06	3.46	41.22
DNA Strand Elongation R-HSA-69190	5 out of 31	1.16E-05	20.02	227.56
Activation Of ATR In Response To Replication Stress R-HSA-176187	5 out of 36	2.47E-05	16.79	178.09
G1/S Transition R-HSA-69206	8 out of 129	3.94E-05	6.96	70.58
Cell Cycle Checkpoints R-HSA-69620	11 out of 271	7.47E-05	4.49	42.70
Mitotic G1 Phase And G1/S Transition R-HSA-453279	8 out of 147	9.92E-05	6.05	55.79
DNA Replication R-HSA-69306	8 out of 155	1.43E-04	5.72	50.63
Synthesis Of DNA R-HSA-69239	7 out of 119	1.68E-04	6.55	56.91
G1/S-Specific Transcription R-HSA-69205	4 out of 29	1.72E-04	16.57	143.63
Unattached Kinetochores Signal Amplification Via A MAD2 Inhibitory Signal R-HSA-141444	6 out of 93	3.00E-04	7.20	58.36
Removal Of Flap Intermediate R-HSA-69166	3 out of 14	3.07E-04	28.12	227.43
EML4 And NUDC In Mitotic Spindle Formation R-HSA-9648025	6 out of 97	3.77E-04	6.88	54.22
Processive Synthesis On Lagging Strand R-HSA-69183	3 out of 15	3.81E-04	25.77	202.90
Mitotic Anaphase R-HSA-68882	9 out of 232	4.70E-04	4.25	32.56
Mitotic Metaphase And Anaphase R-HSA-2555396	9 out of 233	4.85E-04	4.23	32.28
Mitotic Prometaphase R-HSA-68877	8 out of 186	4.92E-04	4.72	35.93
Resolution Of Sister Chromatid Cohesion R-HSA-2500257	6 out of 106	6.05E-04	6.26	46.36
Mitotic Spindle Checkpoint R-HSA-69618	6 out of 110	7.35E-04	6.01	43.39
Metabolism Of Lipids R-HSA-556833	17 out of 732	8.56E-04	2.55	18.01
Lagging Strand Synthesis R-HSA-69186	3 out of 20	9.21E-04	18.19	127.14
S Phase R-HSA-69242	7 out of 161	0.001036	4.75	32.65
RHO GTPases Activate Formins R-HSA-5663220	6 out of 119	0.001109	5.53	37.64
Separation Of Sister Chromatids R-HSA-2467813	7 out of 170	0.001420	4.49	29.42
DNA Replication Pre-Initiation R-HSA-69002	6 out of 127	0.001549	5.16	33.41
Fatty Acid Metabolism R-HSA-8978868	7 out of 173	0.001570	4.41	28.44
CREB1 Phosphorylation Thru NMDA Receptor-Mediated Activation Of RAS Signaling R-HSA-442742	3 out of 27	0.002247	12.88	78.54

Metabolism R-HSA-1430728	33 out of 2049	0.002696	1.80	10.63
FOXO-mediated Transcription Of Oxidative Stress, Metabolic And Neuronal Genes R-HSA-9615017	3 out of 29	0.002767	11.89	70.01
Post NMDA Receptor Activation Events R-HSA-438064	4 out of 61	0.002964	7.26	42.24
Nuclear Events (Kinase And Transcription Factor Activation) R-HSA-198725	4 out of 61	0.002964	7.26	42.24
NR1H2 And NR1H3 Regulate Gene Expression Linked To Lipogenesis R-HSA-9029558	2 out of 9	0.003255	29.31	167.87
Telomere C-strand (Lagging Strand) Synthesis R-HSA-174417	3 out of 33	0.004015	10.30	56.83
Activation Of PPARGC1A (PGC-1alpha) By Phosphorylation R-HSA-2151209	2 out of 10	0.004042	25.64	141.32
Creatine Metabolism R-HSA-71288	2 out of 10	0.004042	25.64	141.32
M Phase R-HSA-68886	10 out of 380	0.004241	2.84	15.51
Orc1 Removal From Chromatin R-HSA-68949	4 out of 69	0.004623	6.36	34.20
Pyrimidine Salvage R-HSA-73614	2 out of 11	0.004909	22.79	121.18
Unwinding Of DNA R-HSA-176974	2 out of 11	0.004909	22.79	121.18
RHO GTPase Effectors R-HSA-195258	8 out of 269	0.005003	3.20	16.97
Signaling By NTRK1 (TRKA) R-HSA-187037	5 out of 114	0.005215	4.76	24.99
Activation Of NMDA Receptors And Postsynaptic Events R-HSA-442755	4 out of 74	0.005927	5.90	30.28
NGF-stimulated Transcription R-HSA-9031628	3 out of 39	0.006444	8.58	43.28
Kinesins R-HSA-983189	3 out of 42	0.007924	7.92	38.31
Leading Strand Synthesis R-HSA-69109	2 out of 14	0.007968	17.09	82.60
Signaling By NTRKs R-HSA-166520	5 out of 132	0.009557	4.08	18.96
SUMOylation Of DNA Replication Proteins R-HSA-4615885	3 out of 45	0.009589	7.35	34.17
Cell Surface Interactions At Vascular Wall R-HSA-202733	5 out of 134	0.010157	4.01	18.42
Hemostasis R-HSA-109582	12 out of 576	0.011128	2.24	10.06
Switching Of Origins To A Post-Replicative State R-HSA-69052	4 out of 90	0.011675	4.80	21.37
Removal Of Flap Intermediate From C-strand R-HSA-174437	2 out of 17	0.011682	13.67	60.84
Extension Of Telomeres R-HSA-180786	3 out of 50	0.012784	6.57	28.64
Processive Synthesis On C-strand Of Telomere R-HSA-174414	2 out of 19	0.014502	12.06	51.06
Ras Activation Upon Ca2+ Influx Thru NMDA Receptor R-HSA-442982	2 out of 19	0.014502	12.06	51.06
Transcription Of E2F Targets Under Negative Control By DREAM Complex R-HSA-1362277	2 out of 19	0.014502	12.06	51.06
G2/M Checkpoints R-HSA-69481	5 out of 148	0.015093	3.62	15.17

Regulation Of Cholesterol Biosynthesis By SREBP (SREBF) R-HSA-1655829	3 out of 55	0.016518	5.94	24.35
PCNA-Dependent Long Patch Base Excision Repair R-HSA-5651801	2 out of 21	0.017584	10.79	43.61
Transcriptional Regulation By MECP2 R-HSA-8986944	3 out of 60	0.020799	5.41	20.97
Nucleotide Salvage R-HSA-8956321	2 out of 23	0.020917	9.76	37.75
Post-chaperonin Tubulin Folding Pathway R-HSA-389977	2 out of 23	0.020917	9.76	37.75
Assembly Of Pre-Replicative Complex R-HSA-68867	4 out of 110	0.022729	3.89	14.73
Polymerase Switching On C-strand Of Telomere R-HSA-174411	2 out of 25	0.024490	8.91	33.06
Resolution Of AP Sites Via Multiple-Nucleotide Patch Replacement Pathway R-HSA-110373	2 out of 25	0.024490	8.91	33.06
FOXO-mediated Transcription R-HSA-9614085	3 out of 65	0.025633	4.98	18.23
RNA Polymerase III Transcription Initiation From Type 2 Promoter R-HSA-76066	2 out of 26	0.026364	8.54	31.05
Formation Of Tubulin Folding Intermediates By CCT/TriC R-HSA-389960	2 out of 26	0.026364	8.54	31.05
HDR Thru Homologous Recombination (HRR) R-HSA-5685942	3 out of 67	0.027720	4.82	17.28
SUMOylation R-HSA-2990846	5 out of 174	0.028053	3.06	10.93
RNA Polymerase III Transcription Initiation From Type 1 Promoter R-HSA-76061	2 out of 27	0.028293	8.20	29.23
Effects Of PIP2 Hydrolysis R-HSA-114508	2 out of 27	0.028293	8.20	29.23
G0 And Early G1 R-HSA-1538133	2 out of 27	0.028293	8.20	29.23
Chromosome Maintenance R-HSA-73886	4 out of 118	0.028473	3.62	12.87
Homology Directed Repair R-HSA-5693538	4 out of 119	0.029247	3.59	12.66
Prefoldin Mediated Transfer Of Substrate To CCT/TriC R-HSA-389957	2 out of 28	0.030277	7.88	27.57
Energy Dependent Regulation Of mTOR By LKB1-AMPK R-HSA-380972	2 out of 29	0.032315	7.59	26.05
Membrane Trafficking R-HSA-199991	11 out of 599	0.033330	1.95	6.65
Regulation Of MECP2 Expression And Activity R-HSA-9022692	2 out of 31	0.036547	7.07	23.38
Sealing Of Nuclear Envelope (NE) By ESCRT-III R-HSA-9668328	2 out of 31	0.036547	7.07	23.38
Gluconeogenesis R-HSA-70263	2 out of 32	0.038738	6.83	22.21
COPI-dependent Golgi-to-ER Retrograde Traffic R-HSA-6811434	3 out of 78	0.040764	4.11	13.15
Cooperation Of Prefoldin And TriC/CCT In Actin And Tubulin Folding R-HSA-389958	2 out of 33	0.040978	6.61	21.12

Factors Involved In Megakaryocyte Development And Platelet Production R-HSA-983231	4 out of 136	0.044301	3.12	9.73
TP53 Regulates Metabolic Genes R-HSA-5628897	3 out of 81	0.044772	3.95	12.27
RNA Polymerase III Transcription Initiation R-HSA-76046	2 out of 35	0.045600	6.21	19.17
Arachidonate Production From DAG R-HSA-426048	1 out of 5	0.047813	25.52	77.58
Regulation Of HMOX1 Expression And Activity R-HSA-9707587	1 out of 5	0.047813	25.52	77.58
Sodium-coupled Phosphate Cotransporters R-HSA-427652	1 out of 5	0.047813	25.52	77.58
Sulfide Oxidation To Sulfate R-HSA-1614517	1 out of 5	0.047813	25.52	77.58
HSF1-dependent Transactivation R-HSA-3371571	2 out of 36	0.047980	6.03	18.30
Mitochondrial Fatty Acid Beta-Oxidation R-HSA-77289	2 out of 36	0.047980	6.03	18.30
Vesicle-mediated Transport R-HSA-5653656	11 out of 637	0.048215	1.83	5.55
Transcriptional Regulation Of White Adipocyte Differentiation R-HSA-381340	3 out of 84	0.048968	3.80	11.48
FU-UR-1 AT3 direct target genes				
Cell Cycle, Mitotic R-HSA-69278	8 out of 523	0.004170	3.346192017	18.33655029
Circadian Clock R-HSA-400253	3 out of 69	0.004730	9.490909091	50.81218251
Mitotic Anaphase R-HSA-68882	5 out of 232	0.005703	4.659892947	24.07688423
Mitotic Metaphase And Anaphase R-HSA-2555396	5 out of 233	0.005805	4.639219015	23.88711048
mTORC1-mediated Signaling R-HSA-166208	2 out of 24	0.006114	18.82575758	95.95691213
Fertilization R-HSA-1187000	2 out of 25	0.006625	18.00634058	90.3361026
Basigin Interactions R-HSA-210991	2 out of 25	0.006625	18.00634058	90.3361026
BMAL1:CLOCK,NPAS2 Activates Circadian Gene Expression R-HSA-1368108	2 out of 27	0.007702	16.56416667	80.60547813
RAB GEFs Exchange GTP For GDP On RABs R-HSA-8876198	3 out of 89	0.009557	7.276376989	33.83853874
Separation Of Sister Chromatids R-HSA-2467813	4 out of 170	0.009802	5.059215586	23.39980205
Activation Of Pre-Replicative Complex R-HSA-68962	2 out of 32	0.010713	13.8	62.60139233
Unattached Kinetochores Signal Amplification Via A MAD2 Inhibitory Signal R-HSA-141444	3 out of 93	0.010769	6.951578947	31.49840393
VEGFA-VEGFR2 Pathway R-HSA-4420097	3 out of 93	0.010769	6.951578947	31.49840393
EML4 And NUDC In Mitotic Spindle Formation R-HSA-9648025	3 out of 97	0.012065	6.654423292	29.39544938
Metabolism Of Vitamins And Cofactors R-HSA-196854	4 out of 186	0.013283	4.61070844	19.92397145
Signaling By VEGF R-HSA-194138	3 out of 102	0.013807	6.316746411	27.0520303

Cell Cycle R-HSA-1640170	8 out of 654	0.014994	2.649604403	11.12862288
Resolution Of Sister Chromatid Cohesion R-HSA-2500257	3 out of 106	0.015298	6.070209504	25.37370356
Mitotic Spindle Checkpoint R-HSA-69618	3 out of 110	0.016877	5.842105263	23.84648518
MTOR Signaling R-HSA-165159	2 out of 41	0.017213	10.61057692	43.10110621
Reproduction R-HSA-1474165	3 out of 113	0.018118	5.681913876	22.78922929
Heme Signaling R-HSA-9707616	2 out of 45	0.020521	9.621608527	37.39261943
RHO GTPases Activate Formins R-HSA-5663220	3 out of 119	0.020751	5.386388385	20.87320759
Rab Regulation Of Trafficking R-HSA-9007101	3 out of 122	0.022142	5.249800973	20.00330253
Metabolism Of Water-Soluble Vitamins And Cofactors R-HSA-196849	3 out of 122	0.022142	5.249800973	20.00330253
Hemostasis R-HSA-109582	7 out of 576	0.023111	2.613627146	9.846718886
Inositol Phosphate Metabolism R-HSA-1483249	2 out of 48	0.023161	8.992753623	33.86022788
Arachidonate Production From DAG R-HSA-426048	1 out of 5	0.024263	51.28350515	190.7125852
Toxicity Of Botulinum Toxin Type D (botD) R-HSA-5250955	1 out of 5	0.024263	51.28350515	190.7125852
Toxicity Of Botulinum Toxin Type F (botF) R-HSA-5250981	1 out of 5	0.024263	51.28350515	190.7125852
Vitamin B1 (Thiamin) Metabolism R-HSA-196819	1 out of 5	0.024263	51.28350515	190.7125852
Regulation Of HMOX1 Expression And Activity R-HSA-9707587	1 out of 5	0.024263	51.28350515	190.7125852
Drug-mediated Inhibition Of CDK4/CDK6 Activity R-HSA-9754119	1 out of 5	0.024263	51.28350515	190.7125852
G1/S Transition R-HSA-69206	3 out of 129	0.025581	4.956390977	18.16966644
Cell Surface Interactions At Vascular Wall R-HSA-202733	3 out of 134	0.028204	4.766010446	17.00657294
PTK6 Regulates Cell Cycle R-HSA-8849470	1 out of 6	0.029046	41.02474227	145.1819826
CD22 Mediated BCR Regulation R-HSA-5690714	1 out of 6	0.029046	41.02474227	145.1819826
Vitamin B2 (Riboflavin) Metabolism R-HSA-196843	1 out of 7	0.033805	34.18556701	115.7918426
Zinc Efflux And Compartmentalization By SLC30 Family R-HSA-435368	1 out of 7	0.033805	34.18556701	115.7918426
Constitutive Signaling By NOTCH1 t(7;9)(NOTCH1:M1580 K2555) Translocation Mutant R-HSA-2660826	1 out of 7	0.033805	34.18556701	115.7918426
Mitotic G1 Phase And G1/S Transition R-HSA-453279	3 out of 147	0.035666	4.332894737	14.44395145
RAB Geranylgeranylation R-HSA-8873719	2 out of 62	0.037157	6.889583333	22.68475283
Axonal Growth Inhibition (RHOA Activation) R-HSA-193634	1 out of 8	0.038541	29.30044183	95.40343353

VEGF Binds To VEGFR Leading To Receptor Dimerization R-HSA-195399	1 out of 8	0.038541	29.30044183	95.40343353
M Phase R-HSA-68886	5 out of 380	0.039066	2.799569892	9.077637199
Sperm Motility And Taxes R-HSA-1300642	1 out of 9	0.043254	25.63659794	80.51615206
Activation Of PUMA And Translocation To Mitochondria R-HSA-139915	1 out of 9	0.043254	25.63659794	80.51615206
p75NTR Regulates Axonogenesis R-HSA-193697	1 out of 9	0.043254	25.63659794	80.51615206
RHO GTPase Effectors R-HSA-195258	4 out of 269	0.043341	3.153271778	9.897072334
Cell Cycle Checkpoints R-HSA-69620	4 out of 271	0.044328	3.129333015	9.751413208
S Phase R-HSA-69242	3 out of 161	0.044726	3.946169221	12.26149807
Orc1 Removal From Chromatin R-HSA-68949	2 out of 69	0.045098	6.167599502	19.11288794
Synthesis Of Pyrophosphates In Cytosol R-HSA-1855167	1 out of 10	0.047944	22.78694158	69.22044886
Glycoprotein Hormones R-HSA-209822	1 out of 10	0.047944	22.78694158	69.22044886
HuR (ELAVL1) Binds And Stabilizes mRNA R-HSA-450520	1 out of 10	0.047944	22.78694158	69.22044886
Interaction With Cumulus Cells And Zona Pellucida R-HSA-2534343	1 out of 10	0.047944	22.78694158	69.22044886
Neurotoxicity Of Clostridium Toxins R-HSA-168799	1 out of 10	0.047944	22.78694158	69.22044886

Supplementary Table 2. Contribution coefficients for genes in PCA plot in Fig. 6g

Gene symbol	PC1 coefficient	PC2 coefficient	PC1 coefficient absolute value
INHBE	-0.370177426	-0.301028375	0.370177426
NEU1	-0.23756408	0.114892569	0.23756408
MIOX	-0.226201812	0.182219044	0.226201812
CDK4	-0.221605632	0.078480517	0.221605632
MET	0.204242236	-0.190206091	0.204242236
BHLHE41	-0.202009127	0.090222659	0.202009127
ADM2	-0.187311597	-0.128412565	0.187311597
RAB3IL1	-0.184383075	-0.050373303	0.184383075
CPVL	-0.181193723	0.076631733	0.181193723
CREB3L1	-0.171689357	-0.040614838	0.171689357
RAB32	-0.169800702	0.06692255	0.169800702
RALGDS	-0.163491216	-0.006658371	0.163491216
CPEB1	-0.154801813	-0.050176279	0.154801813
CUTA	-0.147634547	0.125545792	0.147634547
ID1	-0.144214803	0.144768984	0.144214803
MYEOV	-0.141433162	0.0334767	0.141433162
BAIAP2	-0.137976166	-0.058833466	0.137976166
DNAAF5	-0.136756659	-0.014085813	0.136756659
WWC1	0.136120743	0.044662359	0.136120743
GDF15	-0.135570978	-0.372389726	0.135570978
BIRC7	-0.134164185	0.072546278	0.134164185
PATL1	-0.132084036	0.024830057	0.132084036
BHLHE40	-0.126110289	0.054890496	0.126110289
PRKAG2	-0.118239919	-0.082576269	0.118239919
CHN2	-0.114306268	-0.021319696	0.114306268
CAMKK1	-0.108162896	0.040990243	0.108162896
ATP6V1B2	-0.105905979	-0.007316181	0.105905979
ITPR1	-0.099972804	-0.001607185	0.099972804
SLC25A13	-0.099517538	0.09431633	0.099517538
PLCD3	0.096871775	-0.026859817	0.096871775
SORBS3	-0.095040381	0.080913904	0.095040381
ALYREF	-0.094476586	-0.011427483	0.094476586
VAC14	-0.093091833	0.024137256	0.093091833
METTL8	-0.087959071	0.130630362	0.087959071
PRODH	-0.083462264	0.064666262	0.083462264
BCAR1	0.083397057	-0.009385072	0.083397057
KCP	0.081180388	0.035898894	0.081180388
RHEB	-0.075609707	-0.085094179	0.075609707
ZBED6CL	0.074959588	-0.016805224	0.074959588
KIFC3	0.068814944	0.013333436	0.068814944
EIF4B	0.068610431	-0.049181414	0.068610431
ACTR3C	-0.064483237	-0.067032979	0.064483237
HNRNPF	-0.062059255	0.032376444	0.062059255

MAFG	-0.061336395	-0.146295783	0.061336395
TRIP6	0.060917863	-0.116458171	0.060917863
S100A2	-0.058975353	0.005179431	0.058975353
WBP2	-0.056809905	-0.013018373	0.056809905
GAPDH	-0.054934131	0.008708966	0.054934131
ABCC3	0.054168509	0.1205592	0.054168509
ACBD4	0.052472913	0.035934527	0.052472913
KRT80	0.051047281	-0.123381685	0.051047281
NKAIN4	0.05103473	0.149636179	0.05103473
HES1	0.050819845	-0.103041307	0.050819845

Supplementary Table 3. Key Resources

REAGENT or RESOURCE	SOURCE	IDENTIFIER
Antibodies		
anti-ASPSCR1	Bethyl Laboratories	A302-351A
anti-TFE3	Sigma-Aldrich	HPA023881
anti-TFE3	Cell Signaling Technology	14779S
anti-VCP	Abcam	ab11433
anti-VCP (for ChIP)	Abcam	ab111740
anti-VCP (for ChIP)	Abcam	ab155146
anti-MATR3	Bethyl Laboratories	A300-591A
anti-MTA2	Abcam	ab8106
anti-PRPF8	Abcam	ab79237
anti-H3K27ac	Abcam	ab4729
anti-RNAPOL2	Abcam	ab5131
anti-H3K27ac for native ChiP	mAb from Hiroshi Kimura	[75]
anti-H3K36me3 for native ChiP	Diagenode	C15410192<br>lot#A1857P
anti-H3K4me1 for native ChiP	Diagenode	C15410037<br>lot#A1657D
anti-H3K27me3 for native ChiP	Diagenode	C15410195<br>lot#A1811-001P
anti-GAPDH	Santa Cruz Biotechnology	sc-25778
anti-beta Tubulin	Cell Signaling Technology	2146S
anti-Flag	Sigma-Aldrich	F1804, F3165
anti-FLAG-M2 magnetic beads	Sigma-Aldrich	M8823
anti-EGFP	Abcam	ab184601
anti-RFP	Rockland	600-901-379
anti-LaminB1	Abcam	ab133741
anti-H3	Abcam	ab1791
anti-beta Actin	Thermo Fisher Scientific	AM4302
anti-Ki67 rabbit monoclonal (B56) Alexa Fluor 647	Abcam	ab283699
Alexa fluor 488 anti-rabbit IgG	Thermo Fisher Scientific	A11008
Alexa fluor 594 anti-mouse IgG	Thermo Fisher Scientific	A11032
Alexa fluor 594 anti-rabbit IgG	Thermo Fisher Scientific	A11012
anti-RabbitHRP	Amersham	NA934V
anti-MouseHRP	Cell Signaling Technology	7074S
anti-ChickenHRP	Abcam	Ab6877
Rabbit IgG	Cell Signaling Technology	2729S
Mouse IgG	Santa Cruz Biotechnology	sc-2025
Bacterial and Virus Strains		
DH5α	Thermo Fisher Scientific	18265017
BL21(DE3)RIL	Agilent Technologies	230245
Biological Samples		
Mouse tumors from Rosa26-^{AT3}/$_{CreER}$	generated in lab, ref [5]	
Human tumors confirmed pathologically	HCI, UBC, UCSF,<br>MDACC	
Chemicals, Peptides, and Recombinant Proteins		
siTFE3	Thermo Fisher Scientific	4392420; id#s:<br>14030,14031,14032

siVCP	Thermo Fisher Scientific	4390824; id#s: s14767, s14765
siSCR	Thermo Fisher Scientific	4390844
MISSION 3XLacO inducible pLKO-puro-IPTG-3xLacO lentiviral vectors	Sigma-Aldrich	generated custom for this project
Dynabeads protein G	Thermo Fisher Scientific	10004D
Dynabeads	Invitrogen	M-280
CB-5083	Cayman Chemicals	19311
Laemmli buffer	Santa Cruz Biotechnology	sc-286962
RNAimax	Thermo Fisher Scientific	13778-150
Lipofectamine 3000	Thermo Fisher Scientific	L3000-015
Opti-MEM	Thermo Fisher Scientific	31985-070
Fetal Bovine Serum (FBS)	Thermo Fisher Scientific	10-437-028
Trypsin-EDTA	Thermo Fisher Scientific	25200-056
DMEM (Dulbecco's Modified Eagle Medium)	Thermo Fisher Scientific	11995-065
Ham’s F12 media	Thermo Fisher Scientific	10565-018
IPTG, isopropylβ-D-1-thiogalactopyranoside	Sigma-Aldrich	I6758
D-Luciferin, Potassium Salt	Gold Biotechnology	eLUCK-500
Veriblot	Abcam	ab131366
GoTaq DNA polymerase	Promega	M3008
Platinum Taq DNA polymerase high fidelity	Thermo Fisher Scientific	11304011
Proteinase K	Qiagen	19131
TRIzol reagent	(Ambion) Thermo Fisher	15596018
3XFLAG peptide	Sigma-Aldrich	F4799
GeneRuler DNA ladder mix	Thermo Fisher Scientific	SM0331
1 Kb plus DNA ladder	Thermo Fisher Scientific	10787018
PageRuler plus prestained protein ladder	Thermo Fisher Scientific	26619
SurePAGE, Bis-Tris, Precast gel	Genescript	M00657
Novex wedgewell 4-20% precast gel	Thermo Fisher Scientific	XP04205BOX
RIPA buffer (5X)	Alfa Aesar	J62524
Duolink in situ mounting medium with DAPI	Sigma-Aldrich	DUO82040-5ML
cOmplete, Mini, EDTA-free protease inhibitor cocktail	Roche	4693159001
PhosSTOP, phosphatase inhibitor tablets	Roche	4906837001
PBS	Thermo Fisher Scientific	10010-023
Polybrene	Sigma-Aldrich	TR-1003
Power SYBR green PCR master mix	Thermo Fisher Scientific	4367659
Puromycin dihydrochloride	Sigma-Aldrich	P9620
SuperSignal West Dura Extended Duration Substrate	Thermo Fisher Scientific	34075
TMT10plex™ Isobaric Label Reagent Set	Thermo Fisher Scientific	90110
Trypsin / Lys-C Mix, Mass Spec Grade	Promega	V5071
Tissue-Tek® O.C.T. compound	Sakura	4583
VECTASHIELD antifade mounting medium w/ DAPI	Vector Laboratories	H-1200-10
Duolink® In Situ Red Starter Kit Mouse/Rabbit	MilliporeSigma	DUO92101-1KT
Critical Commercial Assays		
High capacity cDNA synthesis kit	Thermo Fisher Scientific	4374966
Superscript III cDNA synthesis kit	Thermo Fisher Scientific	18080051
Nuclear complex co-IP kit	Active Motif	54001
DNA Clean and Concentrator -5 kit	Zymo Research	D4013
Direct-zol RNA Miniprep Plus kit	Zymo Research	R2071

NEBNExt ChIP-Seq Library Prep with UDI	New England Biolabs	E6440
Illumina TruSeq Stranded mRNA Library Prep w/ UDI	Illumina	20020594
HiChIP Library Prep with UDI	Illumina	
Deposited Data		
Proteomics data from mass spectroscopy	PRIDE database	PXD022515
Genomic datasets from cell lines and mouse tumors	GEO database	GSE162609
Experimental Models: Cell Lines		
FU-UR-1	Marc Ladanyi	
ASPS-1	NCI/NIH	
HEK293T	ATCC	CRL-3216
Experimental Models: Organisms/Strains		
Mus musculus: *Rosa26-LSL-AT3/CreER*	generated in lab	
Mus musculus: NOD.CB17-*Prkdc*^{scid}/NCrCrl	Charles River	Strain #394
Mus musculus: NOD.*Cg-Rag1*^{tm1Mom} *Il2rg*^{tm1Wjl}*/SzJ*	Jackson Laboratories	IMSR_JAX:007799
Oligonucleotides		
RT-PCR primers (listed 5'-to-3')		
18S rRNA-fwd: GGCCCTGTAATTGGAATGAGTC	generated for study	
18S rRNA-rev: CCAAGATCCAACTACGAGCTT	generated for study	
ACTB-fwd: GAGCACAGAGCCTCGCCTTT	generated for study	
ACTB-rev: ACATGCCGGAGCCGTTGTC	generated for study	
ASPSCR1-TFE3-fwd: AAAGAAGTCCAAGTCGGGCCA	generated for study	
ASPSCR1-TFE3-rev: TGGACTCCAGGCTGATGATCTC	generated for study	
ATG9B-fwd: CCAAGACTCACCCATCCAC	generated for study	
ATG9B-rev: AGCTGTAGATCTTGGTGAAGAAA	generated for study	
BHLHE40-fwd: AAAGCACCGGGACTGGA	generated for study	
BHLHE40-rev: CCGTCTTGACTTGTACACTTGG	generated for study	
BHLHE41-fwd: GAGAGACAGTTACTGGAACATAGAG	generated for study	
BHLHE41-rev: CTTGGTGTCGTCTCGTTTCA	generated for study	
CD63-fwd: GCTTCTCTGAACCAGAGTGAC	generated for study	
CD63-rev: AGGAGGACGTAGAGCAAGAA	generated for study	
CTSD-fwd: CAGCCCTCCAGCCTTCT	generated for study	
CTSD-rev: CGGATGGACGTGAACTTG	generated for study	
DBF4-fwd: CCATGAGGATCCACAGTAAAGG	generated for study	
DBF4-rev: AGAGATTTCAGAGATGGTCTGTTT	generated for study	
FAM76B-fwd: CCAAGTGTACCCAGCGTTAT	generated for study	
FAM76B-rev: TGTGCAATCCGACATTCCT	generated for study	
FLCN-fwd: GCAGCTCGTGCAGCTAAG	generated for study	
FLCN-rev: TCGCAGAAGTGGCAGAGA	generated for study	
FNIP2-fwd: CAGGGCTCCTAAGGAAGGA	generated for study	
FNIP2-rev: CTGGTAAACTATCAGGCGAATCT	generated for study	
FZR1-fwd: CTCACCTGTTGATGCGCTAA	generated for study	
FZR1-rev: CTCATTCTGGATGACGATCTGG	generated for study	
GAA-fwd: GTCCGCCCGTTGTTCAG	generated for study	
GAA-rev: TCACTCCCATGGTTGGAGAT	generated for study	
GPNMB-fwd: ATTCAGCATGGAATGTCTCTACT	generated for study	
GPNMB-rev: CTTTCATTGCCCAGCACATC	generated for study	
HIF1A-fwd: GAATATTATCATGCTTTGGACTCTG	generated for study	
HIF1A-rev: GCAAGCATCCTGTACTGTC	generated for study	

HK2-fwd: TCTGCTTGCCTACTTCTTCAC	generated for study	
HK2-rev: ATCTCCAAGAGGGTCTCATCA	generated for study	
HPRT-fwd: ATGGACAGGACTGAACGTCTTGCT	generated for study	
HPRT-rev: TTGAGCACACAGAGGGCTACAATG	generated for study	
IRF2BP2-fwd: AGGCAGGTTGTTGGGTTT	generated for study	
IRF2BP2-rev: GACTTCACCTTCTGGTTCTGG	generated for study	
PEBP1-fwd: CCTTGAGCCTGCAAGAAGT	generated for study	
PEBP1-rev: CGAAATGCTGGTGGGTCTAT	generated for study	
RRAGD-fwd: GGAGGAGGAGGAGGATGAG	generated for study	
RRAGD-rev: CAGGATTCTCGGCTTCACTT	generated for study	
SLC16A1-fwd: AAATCTGGAGGATAGCGTTACA	generated for study	
SLC16A1-rev: GTATCCAACTGGACCTCCAAC	generated for study	
SLC20A1-fwd: GGCTCAGTCAGTGCTATGTT	generated for study	
SLC20A1-rev: CCACGAGGGAGAAACCAATAG	generated for study	
SQSTM1-fwd: CTTCCAGGCGCACTACC	generated for study	
SQSTM1-rev: GTCATCCTTCACGTAGGACAT	generated for study	
UAP1L1-fwd: CTGGAGCGGAAAGACAAAGT	generated for study	
UAP1L1-rev: GGATGTTGTCCACACAGTACA	generated for study	
VCP-fwd: CTTGCCACCGCTCGTAG	generated for study	
VCP-rev: GGGACGGTTCTTCTGTTTGA	generated for study	
WWTR1-fwd: GCTGGGAGATGACCTTCAC	generated for study	
WWTR1-rev: GCTGATTCATCGCCTTCCTA	generated for study	
Bact-fwd: CATTGCTGACAGGATGCAGAAGG mouse	generated for study	
Bact-rev: TGCTGGAAGGTGGACAGTGAGG mouse	generated for study	
Bhlhe40-fwd: TACCTGCCTGCCCAAAG mouse	generated for study	
Bhlhe40-rev: CCTGGACTTGTACACTTGGTA mouse	generated for study	
Bhlhe41-fwd: GAGACAGTTACTGGAACATAGGG m.	generated for study	
Bhlhe41-rev: CTTGGTATCGTCTCGCTTCAA mouse	generated for study	
Cd63-fwd: AGGAGGAATGAAGTGTGTCAAG mouse	generated for study	
Cd63-rev: AAGACAACCTGAACCGCTAC mouse	generated for study	
Cnksr2-fwd: CAAATGGTCTCCGAGTCAAGTA mouse	generated for study	
Cnksr2-rev: TGGTCTCCACTGATCTTCTCT mouse	generated for study	
Ctsd-fwd: GCGTCTTGCTGCTCATTCT mouse	generated for study	
Ctsd-rev: CCGACGGATAGATGTGAACTTG mouse	generated for study	
Dbf4-fwd: GAGGATCCACAGCAAAGCA mouse	generated for study	
Dbf4-rev: GCCTTCAGGGATTTCAAGGA mouse	generated for study	
Eef2k-fwd: CCAGCTCCTTCCACTTCAA mouse	generated for study	
Eef2k-rev: ATGTCCTCGAGATGGAATTCAG mouse	generated for study	
Gpnmb-fwd: CTGCTGGCTGCAGGACT mouse	generated for study	
Gpnmb-rev: TGTGATCGGGATACTGTTCATGG mouse	generated for study	
Grn-fwd: CCCTAGTCCTGGAGCTGAC mouse	generated for study	
Grn-rev: AGCTCATCAGGACCCACAT mouse	generated for study	
Napa-fwd: GGAGCGCAAGGTGAAGAA mouse	generated for study	
Napa-rev: GATCTCGCATGCTTCCTCTATT mouse	generated for study	
Npc2-fwd: CCACGATCCTGCTGCTG mouse	generated for study	
Npc2-rev: GGCTCACATTCACCTCCTTTAT mouse	generated for study	
Slc16a1-fwd: ATGGATATCATCTATAATGTTGGCTGTC mouse	generated for study	
Slc16a1-rev: CCGTATTTATTCACCAAGATACTGCT m.	generated for study	

Slc20a1-fwd: ACGAAACTCAAGATCTGCTCAT mouse	generated for study	
Slc20a1-rev: GGGTCCCAGAAATCGGAAG mouse	generated for study	
Txn1-fwd: AATGGTGAAGCTGATCGAGAG mouse	generated for study	
Txn1-rev: CACACCACGTAGCAGAGAAG mouse	generated for study	
Txn2-fwd: GTCAACAGTGAGACACCAGTT mouse	generated for study	
Txn2-rev: CTGCTTGGCGACCATCTT mouse	generated for study	
Spns1-fwd: CCTCATAGTGGTGGTTCTGTG mouse	generated for study	
Spns1-rev: AGAACTGCTCGATGTCTGTAAG mouse	generated for study	
Uap1l1-fwd: GATCCGCTGTGACCAAGAG mouse	generated for study	
Uap1l1-rev: GCAGGACAGCTACCTTGTT mouse	generated for study	
Vps18-fwd: CCGTCTTGCAGACTGGTT mouse	generated for study	
Vps18-rev: CCGCTGCTTGGTGAAGAT mouse	generated for study	
Genomic DNA qPCR primers (listed 5'-to-3')		
BHLHE40-fwd: TGGAGTCACAGGGTAGAACA	generated for study	
BHLHE40-rev: AAGCCGAGGAGTAATGGAGA	generated for study	
BHLHE41-fwd: GAGAGACAGTTACTGGAACATAGAG	generated for study	
BHLHE41-rev: CTTGGTGTCGTCTCGTTTCA	generated for study	
CBSL-fwd: CACCACAATCCCAGCATACA	generated for study	
CBSL-rev: ATCAAGCCCAGCGAGTTATG	generated for study	
CCND1-fwd: TCCATTCAGAGGTGTGTTTCTC	generated for study	
CCND1-rev: CCTTCATCTTGTCCTTCTAGCC	generated for study	
CCND2-fwd: TCTACCCTACATTCCTGGATCTT	generated for study	
CCND2-rev: CCCTCCAACTTTGGCTTCTT	generated for study	
CDK4-fwd: TCCTACACCTCAGTCCCTAAA	generated for study	
CDK4-rev: GTTATGGAAGGGTCGCTCAA	generated for study	
CTSD-fwd: GCTAGGACAATCAGGAACTGG	generated for study	
CTSD-rev: CAGGAAGCCCAAGACTCAC	generated for study	
DBF4-fwd: ACTGAGAGAGCAACGGAATG	generated for study	
DBF4-rev: CTCGTGCCTGCCTTCTC	generated for study	
DDIT3-fwd: TGCCACTTTCTGATTGGTAGGTT	generated for study	
DDIT3-rev: TGCCACCCGCTCATCTTT	generated for study	
EEF2K-fwd: TTGGTCACTCCCTCGAATTG	generated for study	
EEF2K-rev: GTACACAGTGTCCGGTTCTC	generated for study	
GGA2-fwd: CAGCCTCTCTGATGAGAAACC	generated for study	
GGA2-rev: CGGGCCAGGAGGATAATAATG	generated for study	
GRN-fwd: GCAGGGAGGAGAGTGATTTG	generated for study	
GRN-rev: CGCTCCCATTGGCTACTTAT	generated for study	
NAA10-fwd: CGCTCACCCTCGCATTG	generated for study	
NAA10-rev: GACTGCGCCTTCACGAT	generated for study	
NAPA-fwd: ACCCTGTGGAAGGCAATG	generated for study	
NAPA-rev: CTGCTGATTGGTGGAGGAG	generated for study	
RRAGD-fwd: GGAGGAGGGAGAGAGAGAGA	generated for study	
RRAGD-rev: GGGTGTGTATGTGAGTATGTGAC	generated for study	
SLC16A1-fwd: GCGAGGCTGCCTTATAACC	generated for study	
SLC16A1-rev: GGCTGAAAGCGTGTGGA	generated for study	
SLC20A1-fwd: CTGCAGCAAGGAGTCAGAAT	generated for study	
SLC20A1-rev: GTGGCTGACCTGTGTTTGTA	generated for study	
TXN-fwd: TAACGGTGACCGGGAAGTA	generated for study	
TXN-rev: GAACAGAAGGAGGTTACAGAGAAG	generated for study	

UAP1L1-fwd: GAGTGCCGACTTGACAGAC	generated for study	
UAP1L1-rev: CAGAAGCGCAGGAGGTG	generated for study	
Recombinant DNA		
VCP(wt)-EGFP	AddGene ref [76]	23971
pcDNA-mRFP	AddGene	13032
pIRES2-EGFP	Clontech	6029-1
pET16b	Gift from Chris Hill lab	
Software and Algorithms		
BWA-MEM	ref [56]	version 0.7.10-r789
MACS2	ref [57]	version 2.2.6
MEME	ref [62]	
DeepTools	ref [63]	
R	www.r-project.org	
DeSeq2	ref [65]	version 3.11
featureCounts	ref [64]	version 1.6.3
HOMER	homer.ucsd.edu	
HiC-Pro	ref [68]	
Samtool rmdup	ref [59]	
ChIPpeakAnno	www.bioconductor.org	version 3.22.0
Enrichr	maayanlab.cloud/Enrichr/ refs [66, 67]	
Living Image Software	PerkinElmer IVIS Systems	version 4.7.3

Supplementary References (only cited in the Supplementary Information)

75. Kimura, H., Hayashi-Takanaka, Y., Goto, Y., Takizawa, N. & Nozaki, N. The organization of histone H3 modifications as revealed by a panel of specific monoclonal antibodies. *Cell Struct Funct* **33**, 61-73 (2008).

76. Tresse, E. *et al.* VCP/p97 is essential for maturation of ubiquitin-containing autophagosomes and this function is impaired by mutations that cause IBMPFD. *Autophagy* **6**, 217-227 (2010).
