# Supplementary material for: ASPSCR1-TFE3 reprograms transcription by organizing enhancer loops around hexameric VCP/p97
Source: Nat Commun. 2024 Feb 7;15:1165. doi: 10.1038/s41467-024-45280-5 (PMC10850509; doi:10.1038/s41467-024-45280-5)
Supplement: Supplementary file 3 — Reporting Summary [file 41467_2024_45280_MOESM3_ESM.pdf]

nature portfolio

Corresponding author(s): Kevin B. Jones

Last updated by author(s): Sep 26, 2023

Reporting Summary

Nature Portfolio wishes to improve the reproducibility of the work that we publish. This form provides structure for consistency and transparency in reporting. For further information on Nature Portfolio policies, see our Editorial Policies and the Editorial Policy Checklist.

Statistics

For all statistical analyses, confirm that the following items are present in the figure legend, table legend, main text, or Methods section.

n/a	Confirmed	
☐	☒	The exact sample size (*n*) for each experimental group/condition, given as a discrete number and unit of measurement
☐	☒	A statement on whether measurements were taken from distinct samples or whether the same sample was measured repeatedly
☐	☒	The statistical test(s) used AND whether they are one- or two-sided<br>*Only common tests should be described solely by name; describe more complex techniques in the Methods section.*
☐	☒	A description of all covariates tested
☐	☒	A description of any assumptions or corrections, such as tests of normality and adjustment for multiple comparisons
☐	☒	A full description of the statistical parameters including central tendency (e.g. means) or other basic estimates (e.g. regression coefficient) AND variation (e.g. standard deviation) or associated estimates of uncertainty (e.g. confidence intervals)
☐	☒	For null hypothesis testing, the test statistic (e.g. *F*, *t*, *r*) with confidence intervals, effect sizes, degrees of freedom and *P* value noted<br>*Give P values as exact values whenever suitable.*
☒	☐	For Bayesian analysis, information on the choice of priors and Markov chain Monte Carlo settings
☒	☐	For hierarchical and complex designs, identification of the appropriate level for tests and full reporting of outcomes
☒	☐	Estimates of effect sizes (e.g. Cohen's *d*, Pearson's *r*), indicating how they were calculated

Our web collection on statistics for biologists contains articles on many of the points above.

Software and code

Policy information about availability of computer code

Data collection	Living Image Software PerkinElmer IVIS Systems version 4.7.3.
Data analysis	Graphpad Prism 7.0 software was used for basic statistical tests. Specific software for genomic analyses are listed in the methods pertinent to each section. BWA-MEM version 0.7.10-r789; MACS2 version 2.2.6; MEME; DeepTools; R www.r-project.org; DeSeq2 version 3.11; featureCounts version 1.6.3; HOMER homer.ucsd.edu; HiC-Pro; Samtool rmdup; ChIPpeakAnno version 3.22.0; Enrichr maayanlab.cloud/Enrichr/

For manuscripts utilizing custom algorithms or software that are central to the research but not yet described in published literature, software must be made available to editors and reviewers. We strongly encourage code deposition in a community repository (e.g. GitHub). See the Nature Portfolio guidelines for submitting code & software for further information.

Data

Policy information about availability of data

All manuscripts must include a data availability statement. This statement should provide the following information, where applicable:

- Accession codes, unique identifiers, or web links for publicly available datasets
- A description of any restrictions on data availability
- For clinical datasets or third party data, please ensure that the statement adheres to our policy

The genomics datasets generated in this study are available at GSE162609 on the Gene Expression Omnibus. The raw data genomics data from one patient are protected and are not available due to privacy laws, but processed data are included in the GEO deposit, along with all other raw and processed data.

The proteomics datasets generated in this study are available at PXD022515 on the PRIDE database.

Research involving human participants, their data, or biological material

Policy information about studies with human participants or human data. See also policy information about sex, gender (identity/presentation), and sexual orientation and race, ethnicity and racism.

Reporting on sex and gender	The term sex was used, not gender. Male and female sex is listed for samples, cell lines, and mouse hosts of xenografts.
Reporting on race, ethnicity, or other socially relevant groupings	N/A
Population characteristics	N/A
Recruitment	N/A
Ethics oversight	The University of Utah IRB judged our work with deidentified human specimens to be exempt from formal review.

Note that full information on the approval of the study protocol must also be provided in the manuscript.

Field-specific reporting

Please select the one below that is the best fit for your research. If you are not sure, read the appropriate sections before making your selection.

☒ Life sciences ☐ Behavioural & social sciences ☐ Ecological, evolutionary & environmental sciences

For a reference copy of the document with all sections, see nature.com/documents/nr-reporting-summary-flat.pdf

Life sciences study design

All studies must disclose on these points even when the disclosure is negative.

Sample size	Sample sizes are explained throughout the manuscript. Power analyses for mouse experiments identified n=10 as a goal inclusion per group.
Data exclusions	No data were excluded from analyses.
Replication	Most experiments were replicated across multiple model systems as demonstrated in the data presented. Technical replication of experiments is noted in Figure legends, when applicable.
Randomization	The grouping of xenografted human tumors in mice or mice developing tumors spontaneously was done by randomization as explained in the text. Cell culture experiments were performed with same passage controls done in parallel for each replication of the experiment.
Blinding	For all pathological assessments, the reviews of tissues samples were performed in blinded fashion, as noted. Blinding is built-in to all other assessments of genomic datasets.

Reporting for specific materials, systems and methods

We require information from authors about some types of materials, experimental systems and methods used in many studies. Here, indicate whether each material, system or method listed is relevant to your study. If you are not sure if a list item applies to your research, read the appropriate section before selecting a response.

Materials & experimental systems

n/a	Involved in the study	
☐	☒	Antibodies
☐	☒	Eukaryotic cell lines
☒	☐	Palaeontology and archaeology
☐	☒	Animals and other organisms
☒	☐	Clinical data
☒	☐	Dual use research of concern
☒	☐	Plants

Methods

n/a	Involved in the study	
☐	☒	ChIP-seq
☐	☒	Flow cytometry
☒	☐	MRI-based neuroimaging

Antibodies

Antibodies used	Antibodies<br>anti-ASPSCR1 Bethyl Laboratories A302-351A<br>anti-TFE3 Sigma-Aldrich HPA023881<br>anti-TFE3 Cell Signaling Technology 14779S<br>anti-VCP Abcam ab11433<br>anti-VCP (for ChIP) Abcam ab111740<br>anti-VCP (for ChIP) Abcam ab155146<br>anti-MATR3 Bethyl Laboratories A300-591A<br>anti-MTA2 Abcam ab8106<br>anti-PRPF8 Abcam ab79237<br>anti-H3K27ac Abcam ab4729<br>anti-RNAPOL2 Abcam ab5131<br>anti-H3K27ac for native ChiP mAb from Hiroshi Kimura 27<br>anti-H3K36me3 for native ChiP Diagenode C15410192 lot#A1857P<br>anti-H3K4me1 for native ChiP Diagenode C15410037 lot#A1657D<br>anti-H3K27me3 for native ChiP Diagenode C15410195 lot#A1811-001P<br>anti-GAPDH Santa Cruz Biotechnology sc-25778<br>anti-beta Tubulin Cell Signaling Technology 2146S<br>anti-Flag Sigma-Aldrich F1804, F3165<br>anti-FLAG-M2 magnetic beads Sigma-Aldrich M8823<br>anti-EGFP Abcam ab184601<br>anti-RFP Rockland 600-901-379<br>anti-LaminB1 Abcam ab133741<br>anti-H3 Abcam ab1791<br>anti-beta Actin Thermo Fisher Scientific AM4302<br>Alexa fluor 488 anti-rabbit IgG Thermo Fisher Scientific A11008<br>Alexa fluor 594 anti-mouse IgG Thermo Fisher Scientific A11032<br>Alexa fluor 594 anti-rabbit IgG Thermo Fisher Scientific A11012<br>anti-RabbitHRP Amersham NA934V<br>anti-MouseHRP Cell Signaling Technology 7074S<br>anti-ChickenHRP Abcam Ab6877<br>Rabbit IgG Cell Signaling Technology 2729S<br>Mouse IgG Santa Cruz Biotechnology sc-2025
Validation	Antibodies were validated internally by testing for appropriately sized proteins by western blot and cross reaction with alternative antibodies to each epitope or another portion of the same protein. Each also came with manufacturer validations noted on the respective websites.

Eukaryotic cell lines

Policy information about cell lines and Sex and Gender in Research

Cell line source(s)	HEK293T cells were obtained from ATCC and were maintained in DMEM supplemented with 10% FBS.<br>FU-UR-1 cells were obtained from Marc Ladanyi at Memorial Sloan-Kettering and were maintained in Dulbecco's Modified Eagle Medium (DMEM) plus Ham's F12 media (1:1 volumetric ratio) supplemented with 10% FBS. This is a male cell line.<br>ASPS-1 cells were obtained from the Division of Cancer Treatment and Diagnosis, NCI, NIH, USA and were maintained in DMEM/F12 (1:1 ratio) media supplemented with 10% FBS. This is a female cell line.
Authentication	Each cell line was tested at least annually by STR sequencing to confirm stability and identity of the cells in prolonged culture.
Mycoplasma contamination	All cells were tested for mycoplasma contamination and found to be clean when brought into the laboratory.
Commonly misidentified lines<br>(See ICLAC register)	No commonly misidentified cell lines were used in the study.

Animals and other research organisms

Policy information about studies involving animals; ARRIVE guidelines recommended for reporting animal research, and Sex and Gender in Research

Laboratory animals	Mus musculus was used, mixed strain C57Bl/6-svJ aged 2-6 months, NRG aged 2-4 months,.
Wild animals	No wild animals were used in the study.
Reporting on sex	Male and female mice were noted for each experiment, most experiments including both.
Field-collected samples	No field-collected samples were used in the study.

Ethics oversight | All mouse experiments were performed under the auspices of the Institutional Animal Care and Use Committees at the University of Utah or the University of Calgary and in accordance with international legal and ethical guidelines.

Note that full information on the approval of the study protocol must also be provided in the manuscript.

Plants

Seed stocks: N/A

Novel plant genotypes: N/A

Authentication: N/A

ChIP-seq

Data deposition

☒ Confirm that both raw and final processed data have been deposited in a public database such as GEO.

☒ Confirm that you have deposited or provided access to graph files (e.g. BED files) for the called peaks.

Data access links
May remain private before publication.

RNA-seq, ChIP-seq, and HiChIP sequencing data are on the GEO.
Accession: GSE162609 is the SuperSeries, with 14 SubSeries included
Reviewer Access: https://www.ncbi.nlm.nih.gov/geo/query/acc.cgi?acc=GSE162609
Token: ajglmquypziddah

Files in database submission

GSE162068 - ASPSCR1-TFE3 reprograms transcription by organizing enhancers around hexameric VCP [HiChIP]
GSE162476 - ASPSCR1-TFE3 reprograms transcription by organizing enhancers around hexameric VCP [mASPS_ChIP, ASPL_VCP_Pol2]
GSE162477 - ASPSCR1-TFE3 reprograms transcription by organizing enhancers around hexameric VCP [mASPS_ChIP, histone_markers]
GSE162478 - ASPSCR1-TFE3 reprograms transcription by organizing enhancers around hexameric VCP [hASPS_ChIP, ASPL_VCP_Pol2]
GSE162479 - ASPSCR1-TFE3 reprograms transcription by organizing enhancers around hexameric VCP [hASPS_ChIP, histone_markers]
GSE162480 - ASPSCR1-TFE3 reprograms transcription by organizing enhancers around hexameric VCP [Cell_lines_ChIP, FUUR1, 17934_FUUR1_KDs_H3K27ac]
GSE162481 - ASPSCR1-TFE3 reprograms transcription by organizing enhancers around hexameric VCP [Cell_lines_ChIP, FUUR1, FUUR1_ASPL_POLII]
GSE162482 - ASPSCR1-TFE3 reprograms transcription by organizing enhancers around hexameric VCP [Cell_lines_ChIP, FUUR1, FUUR1_VCP]
GSE162483 - ASPSCR1-TFE3 reprograms transcription by organizing enhancers around hexameric VCP [Cell_lines_ChIP, ASPS1]
GSE162486 - ASPSCR1-TFE3 reprograms transcription by organizing enhancers around hexameric VCP [RNA-Seq, ASPS1_KD]
GSE162488 - ASPSCR1-TFE3 reprograms transcription by organizing enhancers around hexameric VCP [RNA-Seq, FUUR1_KD]
GSE162490 - ASPSCR1-TFE3 reprograms transcription by organizing enhancers around hexameric VCP [RNA-Seq, FUUR_CB5083]
GSE162575 - ASPSCR1-TFE3 reprograms transcription by organizing enhancers around hexameric VCP [RNA-Seq, 17637_HEK_cdelta]
GSE162576 - ASPSCR1-TFE3 reprograms transcription by organizing enhancers around hexameric VCP [RNA-Seq, ASPS1_CB5083]
GSE162609 - ASPSCR1-TFE3 reprograms transcription by organizing enhancers around hexameric VCP
GSE162727 - ASPSCR1-TFE3 reprograms transcription by organizing enhancers around hexameric VCP [ASPS1 VCP ChIP]

Genome browser session
(e.g. UCSC)

no longer applicable

Methodology

Replicates

FUUR1(VCP, ASPL, RNAPol2): 4
ASPS1(VCP, ASPL, RNAPol2): 3
Human tumors (VCP, ASPL, RNAPol2): 2
Human tumors (H3K27ac, H3K27me3, H3K4me1, H3K36me3):2
Mouse tumors (VCP, ASPL, RNAPol2): 2
Mouse tumors (H3K27ac, H3K27me3, H3K4me1, H3K36me3):5

Sequencing depth: 2X50 bp paired-end ~50 M reads/sample

Antibodies	anti-ASPSCR1 Bethyl Laboratories A302-351A<br>anti-VCP (for ChIP) Abcam ab111740<br>anti-VCP (for ChIP) Abcam ab155146<br>anti-H3K27ac Abcam ab4729<br>anti-RNAPOL2 Abcam ab5131<br>anti-H3K27ac for native ChiP mAb from Hiroshi Kimura 32<br>anti-H3K36me3 for native ChiP Diagenode C15410192 lot#A1857P<br>anti-H3K4me1 for native ChiP Diagenode C15410037 lot#A1657D<br>anti-H3K27me3 for native ChiP Diagenode C15410195 lot#A1811-001P
Peak calling parameters	Aligments: Novoagliner<br>Mouse: mm10.standard.nov.illumina.nix<br>Human: hg38.nov.illumina.nix;<br>Peakcalling Software: MACS2 (parameters: callpeak -B --SPMR --qvalue=1e-3 --mfold 15 100)
Data quality	All raw reads are subject to QC pipeline with FastQC package, which correct the issue of position-dependent biases ("Per base sequence quality" analysis), sequencing adapter contamination ("Overrepresented sequences" analysis), or DNA over amplification ("Sequence duplication levels" analysis). A further Quality control is performed in mapping step, to remove duplicates, low mapping quality alignments (< 30), check chromosome content and insert size (~200-300 bp). We finally Visualize mapped data in genome browsers (e.g., IGV) to manually inspect alignments and check for any anomalies or misalignments.
Software	Mapping with Novoaligner. Peakcalling with MACS2 (2.2.7.1).<br>Visualization in IGV (2.16.2).<br>Peaks annotation are done by ChIPseeker (1.26.2).<br>Replicates correlation and Enrichment heatmap are performed with Deeptools (3.5.1)<br>Peaks operation mostly are done by bedtools (v2.29.2)

Flow Cytometry

Plots

Confirm that:

☒ The axis labels state the marker and fluorochrome used (e.g. CD4-FITC).

☒ The axis scales are clearly visible. Include numbers along axes only for bottom left plot of group (a 'group' is an analysis of identical markers).

☒ All plots are contour plots with outliers or pseudocolor plots.

☒ A numerical value for number of cells or percentage (with statistics) is provided.

Methodology

Sample preparation	FU-UR-1 cells were treated with 0.5 µM of CB5083 or DMSO for 72 h at 37°C with 5% CO2. Also, FU-UR-1 cells were treated with 0.5 uM of CB5083 or DMSO in combination with siRNA transfection using Invitrogen Lipofectamine RNAiMAX Transfection Reagent for 72 h at 37°C with 5% CO2. Mouse embryonic fibroblasts (MEFs) were treated with 10 mg/ml TAT-Cre (Excellgen) and/or transfected with CMV-GFP or CMV-VCP-GFP expression vectors using Invitrogen Lipofectamine 3000 Transfection Reagent and incubated for 72 h at 37°C with 5% CO2. For cell proliferation analyses all the samples were prepared according to the manufacturer's protocol using Invitrogen CellTraceTM Cell Proliferation Kit. In brief, after sample treatment, the culture medium was removed from the cells and replaced with a loading solution (1 µM CellTraceTM Far Red dye or 5 µM CellTraceTM Yellow dye in pre-warmed PBS (37°C)). Cells were incubated for 20 minutes at 37°C. Following loading solution removal, cells were washed twice with pre-warmed complete culture medium, and incubated for at least 10 minutes with fresh, pre-warmed complete culture medium. The cells were harvested from the 60 mm dishes by trypsinization, centrifuged at 300 rpm for 3 minutes, resuspended with PBS, and proceeded to analysis.
Instrument	CytoFLEX LX
Software	BD FACSDiva 8.0
Cell population abundance	5000 (Fig.7a) or 20000 (Fig. 7c-d) FU-UR-1 cells were taken for each sample. 5000 MEFs were taken for each sample.
Gating strategy	To obtain the population of interest, rare debris and cell doublets were removed using FSC vs. SSC plots.

☐ Tick this box to confirm that a figure exemplifying the gating strategy is provided in the Supplementary Information.
