# Supplementary figures and images for: ASPSCR1-TFE3 reprograms transcription by organizing enhancer loops around hexameric VCP/p97
Source: Nat Commun. 2024 Feb 7;15:1165. doi: 10.1038/s41467-024-45280-5 (PMC10850509; doi:10.1038/s41467-024-45280-5)

Figure 1b whole blots

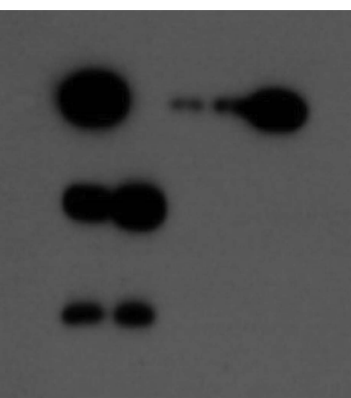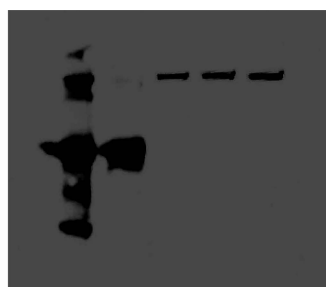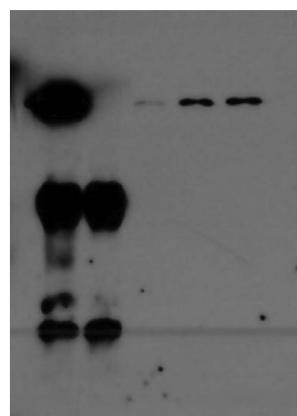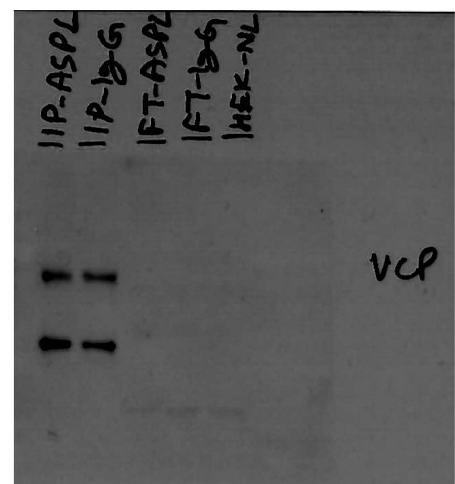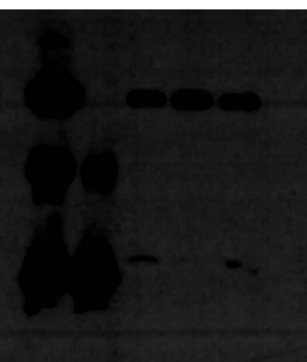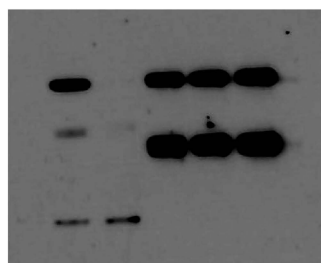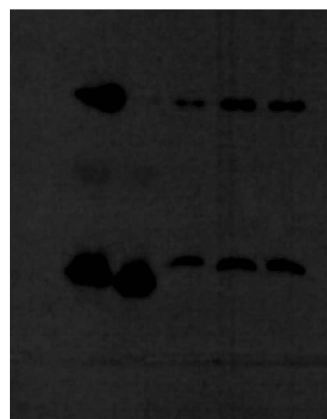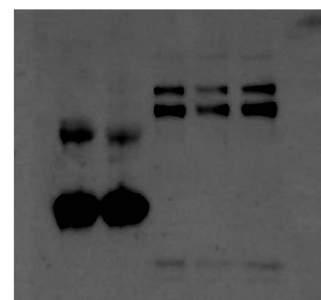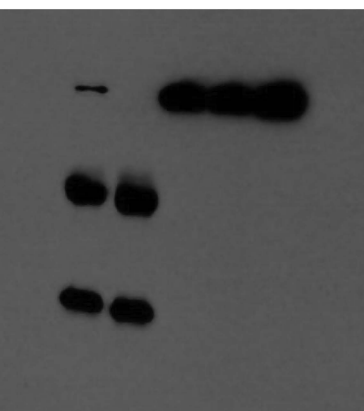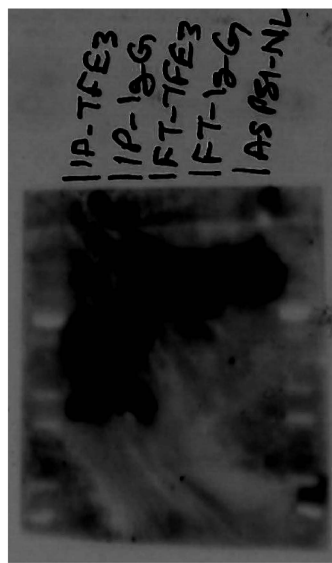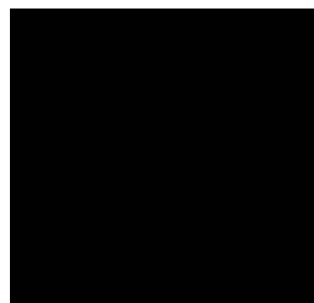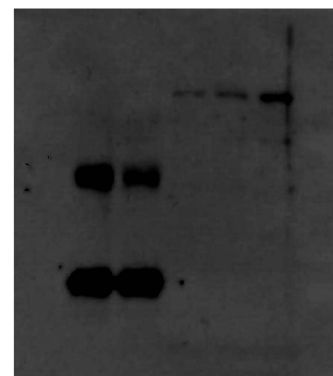

Figure 1c whole blots

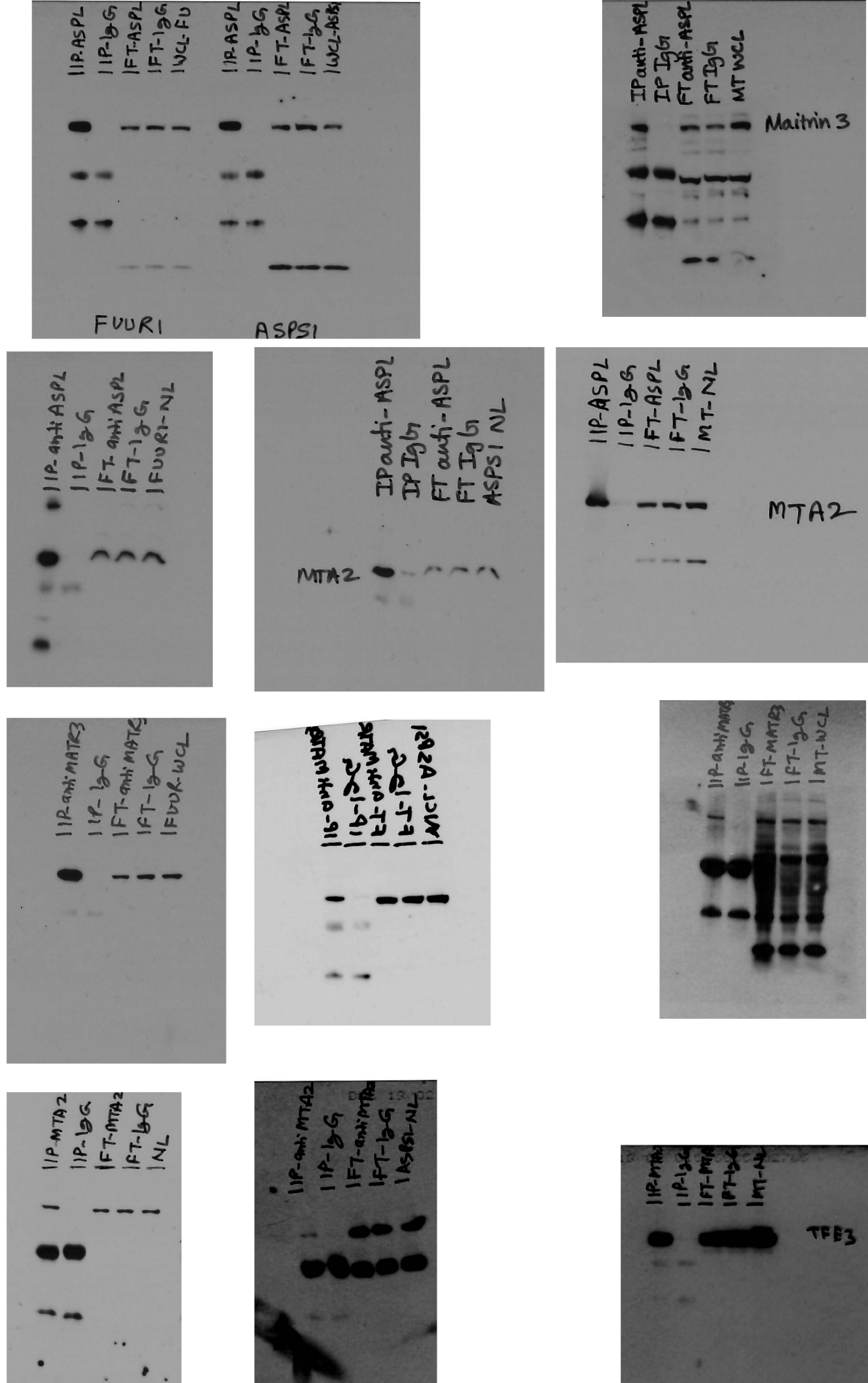

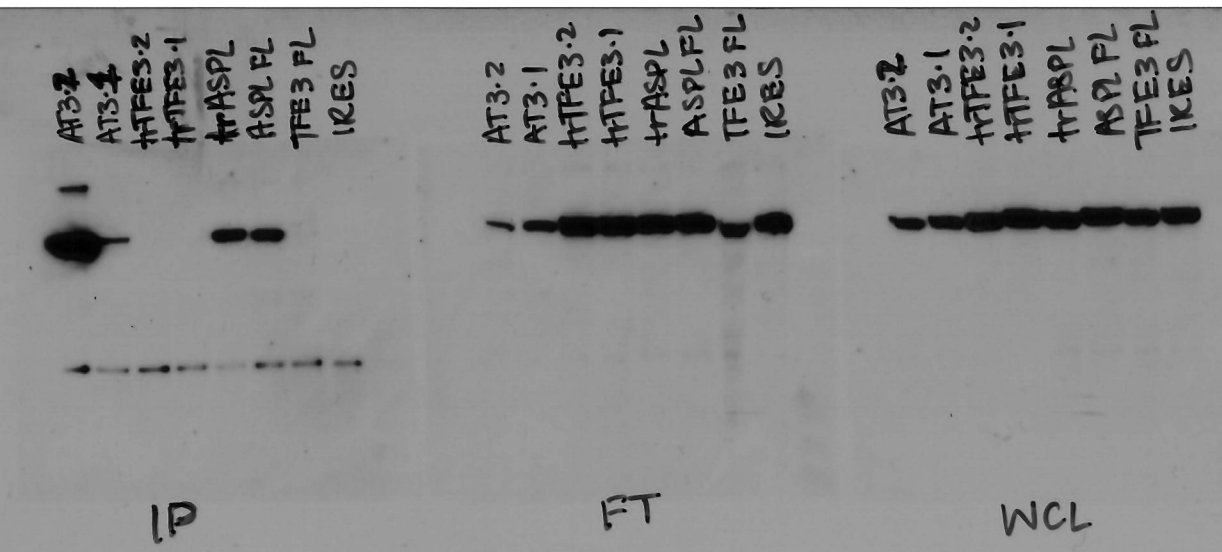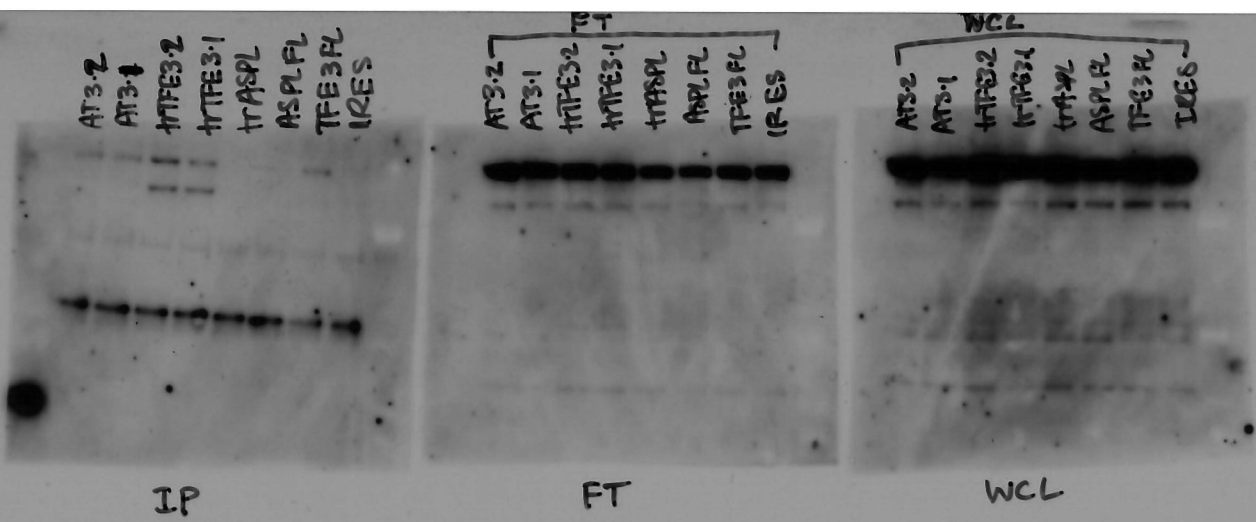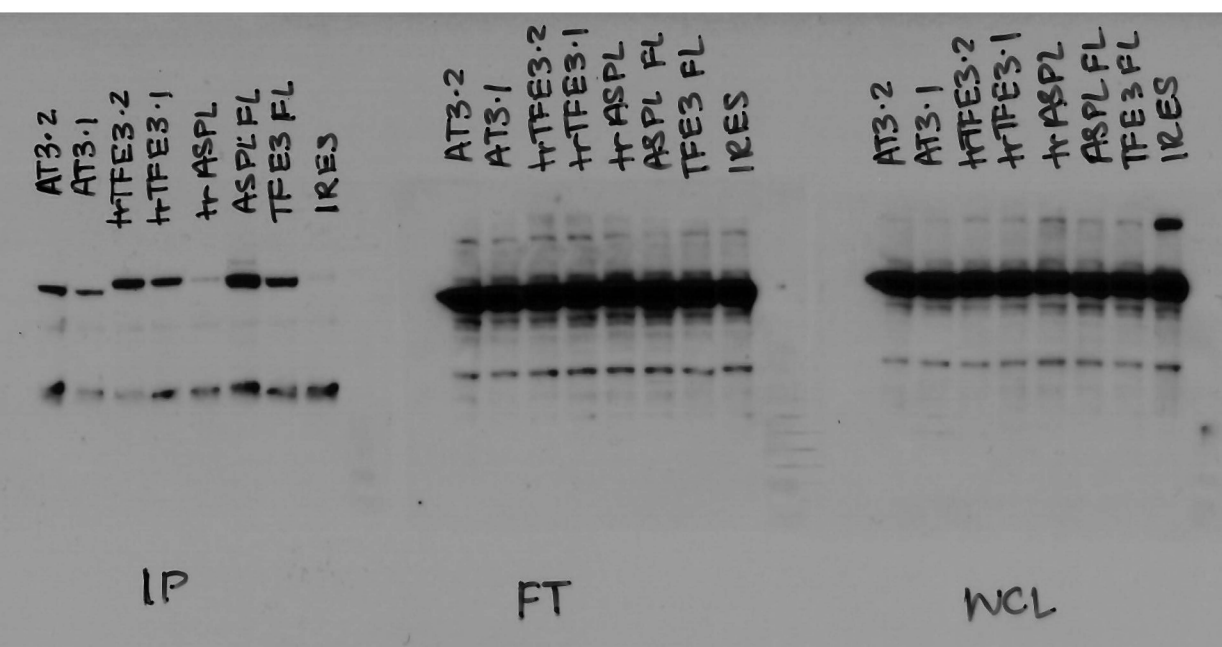

Figure 1g whole blots

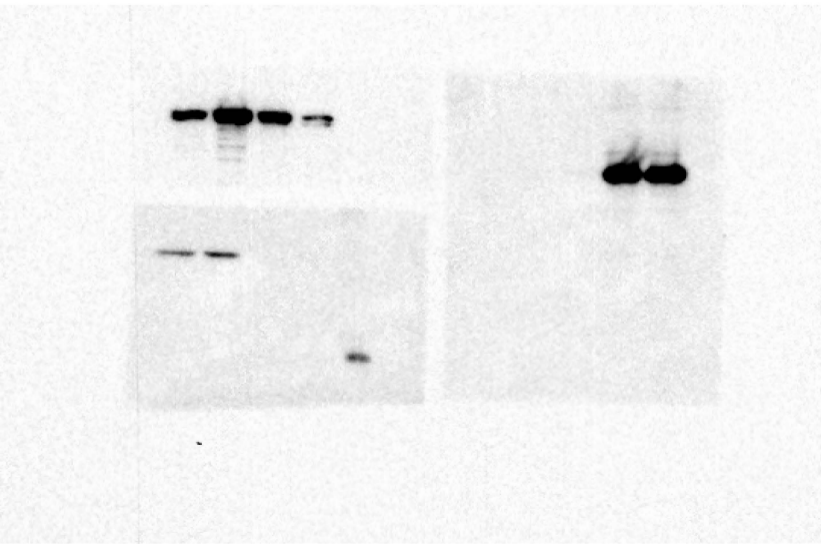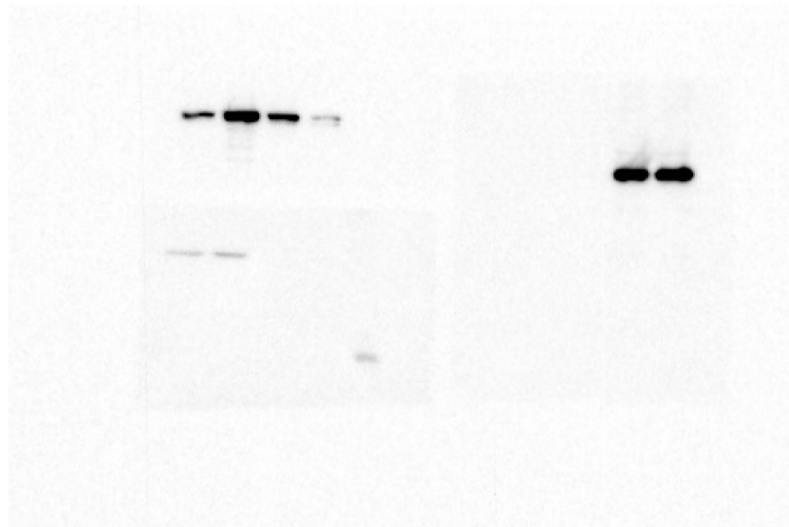

Supplement: Supplementary file 4 — Source Data [file 41467_2024_45280_MOESM4_ESM.zip › Source Data/UncroppedScansVCPinASPS.pdf]
